# ﻿A review of the cavernicolous Trichopolydesmidae (Diplopoda, Polydesmida) from the Carpathian-Balkan arch and the Rhodope Mountains, with descriptions of two new genera and three new species

**DOI:** 10.3897/zookeys.1097.83916

**Published:** 2022-04-19

**Authors:** Dragan Antić, Boyan Vagalinski, Pavel Stoev, Nesrine Akkari

**Affiliations:** 1 University of Belgrade–Faculty of Biology, Institute of Zoology, Studentski trg 16, 11000 Belgrade, Serbia; 2 Serbian Biospeleological Society, Trg Dositeja Obradovića 2, 21000 Novi Sad, Serbia; 3 Institute of Biodiversity and Ecosystem Research at the Bulgarian Academy of Sciences, 2 Yurii Gagarin Street, 1113, Sofia, Bulgaria; 4 National Museum of Natural History, Bulgarian Academy of Sciences, Tsar Osvoboditel Blvd. 1, Sofia 1000, Bulgaria; 5 Pensoft Publishers, Sofia, Bulgaria; 6 3; 7 rd; 8 Zoological Department, Natural History Museum Vienna, Burgring 7, 1010 Vienna, Austria

**Keywords:** Balkan Peninsula, Bulgaria, caves, millipedes, new combination, new status, Serbia, taxonomy

## Abstract

All cavernicolous species of the millipede family Trichopolydesmidae from the Carpathian-Balkan arch and the Rhodope Mountains have been reviewed. At present the family has been shown to comprise five or six genera with eight or nine species. Two new genera have been described, viz., *Balkanodesminus***gen. nov.**, with two new species: *B.dentatoides***sp. nov.** and *B.serbicus***sp. nov.**, from Bulgaria and Serbia, respectively, and the monospecific *Rhodopodesmus***gen. nov.**, with *R.niveus***sp. nov.**, from Bulgaria. Two new combinations and one new status have been proposed: *Balkanodesminusbulgaricus* (Strasser, 1962) **comb. nov.** ex *Bacillidesmusbulgaricus* Strasser, 1962 and *Balkanodesminusdentatus* (Strasser, 1966a) **comb. nov.**, **stat. nov.** ex *Bacillidesmusbulgaricusdentatus* Strasser, 1966a. All genera and species are diagnosed with the inclusion of the most relevant remarks for each of them. Old museum types are checked for *Bacillidesmusfiliformis* (Latzel, 1884) with lectotype designation, as well as for *Trichopolydesmuseremitis* Verhoeff, 1898. An identification key to all six genera and a distribution map for the eight species are provided, as well as brief remarks and general considerations on the family Trichopolydesmidae.

## ﻿Introduction

The type species of the family Trichopolydesmidae, *Trichopolydesmuseremitis* Verhoeff, 1898 was described based on a single male collected in a cave near Băile Herculane in Romania. Given the limited general knowledge on the millipedes of that time, Verhoeff (1898) stated that the genus *Trichopolydesmus* Verhoeff, 1898 could in some respects be related to the genus *Strongylosoma* Brandt, 1833 (today in Paradoxosomatidae). At the same time, based on Latzel’s (1884) specimens of the species *Brachydesmusfiliformis* Latzel, 1884, Attems (1898) erected a new genus, *Bacillidesmus* Attems, 1898, which is today considered to be closely related to *Trichopolydesmus*. For these two genera, Verhoeff (1910) created two monospecific subfamilies within the family Polydesmidae, viz., Trichopolydesminae and Bacillidesminae. Brölemann (1916), to a certain extent, accepted Verhoeff’s (1910) higher taxonomic ranking of the aforementioned taxon, but considered it as the tribe Trichopolydesmini, to which he assigned several other European and North African genera (mostly from the Mediterranean region). Later on, Attems (1926, 1940) considered *Trichopolydesmus* and *Bacillidesmus* as members of the family Vanhoeffeniidae, an opinion with which Verhoeff (1941b) largely disagreed, considering Vanhoeffeniidae unacceptably heterogeneous. In that same paper he (Verhoeff 1941b) put the genus *Trichopolydesmus*, together with some South American taxa, in its own family, Trichopolydesmidae, not taking into consideration Brölemann’s (1916) earlier classification of the tribe Trichopolydesmini. In the same work, Verhoeff (1941b) erected the monospecific family Bacillidesmidae for the genus *Bacillidesmus*. As for the family Vanhoeffeniidae, Jeekel (1956) argued that its type genus *Vanhoeffenia* Attems, 1908 (see Attems 1908) is rather a member of the family Sphaerotrichopodidae, thus suppressing the family Vanhoeffeniidae. This act was apparently missed by some authors (e.g., Ceuca 1958 and Schubart 1960) who continued using the name Vanhoeffeniidae. Some years later, Jeekel (1965) synonymized Sphaerotrichopodidae and Vanhoeffeniidae under Dalodesmidae.

In the second half of the 20^th^ century, the status and the composition of Trichopolydesmidae remained debatable. Ribaut (1955) followed Brölemann’s (1916) vision and included *Galliocookia* Ribaut, 1955 in the tribe Trichopolydesmini. Tabacaru (1975, 1980) treated the family Trichopolydesmidae in Verhoeff’s (1941b) sense, with some South American taxa, but focused only on European taxa, and besides *Trichopolydesmus*, he added some other taxa, including *Bacillidesmus*. Hoffman (1980) restricted the family to only a few European genera. This concept was more or less followed by Mauriès (1984) who put in the family several European genera sensu Tabacaru (1975, 1980) and Hoffman (1980), one North African genus sensu Brölemann (1916), as well as several other European genera. Thus, considering Trichopolydesmidae to comprise taxa with chiefly Mediterranean distributions. Golovatch (2011) followed Mauriès’ (1984) classification and additionally assigned to it the genus *Caucasodesmus* Golovatch, 1985 from the Caucasus and the Crimean Peninsula.

As far as the higher classification is concerned, Hoffman (1980) recognized the superfamily Trichopolydesmoidea within the suborder Polydesmidea. According to the same author, this superfamily includes all taxa that once belonged to the family Vanhoeffeniidae. He (Hoffman 1980) classified them into four families, viz., Trichopolydesmidae, Macrosternodesmidae, Nearctodesmidae and Fuhrmannodesmidae. In addition to these four families, Golovatch (2011) added the small Mediterranean family Mastigonodesmidae, simultaneously sharing Hofmann’s (1980) view that the Fuhrmannodesmidae is a very heterogeneous family and that its members need to be divided into several natural groups. Two years later, Golovatch et al. (2013) further included in the group the family Opisotretidae, which had earlier been classified in the superfamily Polydesmoidea (sensu Hoffman 1980) or in its own superfamily Opisotretoidea (sensu Simonsen 1990). Interestingly, in the same year, Golovatch (2013) synonymized the families Mastigonodesmidae, Macrosternodesmidae, Nearctodesmidae, and Fuhrmannodesmidae with the family Trichopolydesmidae, leaving the Trichopolydesmoidea with only two families, viz., Trichopolydesmidae and Opisotretidae. In this way, Trichopolydesmidae became a large and obviously very heterogeneous group of millipedes. This view of the family Trichopolydesmidae was not well accepted by other authors, primarily due to the lack of a good diagnosis of this group (Antić et al. 2014; Tabacaru and Giurginca 2016). Tabacaru and Giurginca (2016) largely disagreed with such a classification of Trichopolydesmidae and restricted it to the European taxa only (12 genera), with the family’s distribution spanning from the Iberian Peninsula, through the Alps, the Balkans, the Aegean region, the Crimean Peninsula all the way to the North Caucasus. A disagreement with Golovatch’s (2013) classification was also expressed by Shear and Reddell (2017). These authors excluded the families Macrosternodesmidae and Nearctodesmidae from Trichopolydesmidae, leaving Macrosternodesmidae as a separate family with two subfamilies, Macrosternodesminae and Nearctodesminae, simultaneously synonymizing the superfamily Trichopolydesmoidea under Polydesmoidea. Finally, Golovatch et al. (2018, 2022), obviously accepted this act by Shear and Reddell (2017), but still treated Trichopolydesmidae in a broader sense, including Fuhrmannodesmidae and Mastigonodesmidae therein, with > 220 species in approximately 100 genera.

In the present paper, we review the cavernicolous members of the millipede family Trichopolydesmidae in the Carpathian-Balkan arch and the Rhodope Mountains (stretched between Bulgaria and Greece) and demonstrate that its fauna contains five or six genera with eight or nine species, including two genera and three species described here as new.

## ﻿Material and methods

### ﻿Preservation, dissecting, imaging, map

Specimens preserved in 70% ethanol were examined with a Nikon SMZ 745T and a Zeiss Stemi 2000-C binocular stereo microscopes (IZB), a Nikon SMZ25 stereo microscope (NHMW), or a Carl Zeiss Discovery V8 stereo microscope (Institute of Biodiversity and Ecosystem Research). The gonopods and legs were dissected and mounted in glycerin for temporary microscope preparations and observed with a Carl Zeiss Axioscope 40 microscope (IZB). The gonopod and legs of *Bacillidesmusfiliformis* type specimens, as well as habitus and gonopod of *Trichopolydesmuseremitis* holotype were photographed with a DS-Ri-2 camera mounted on a Nikon Eclipse Ni microscope using NIS-Elements Microscope Imaging Software with an Extended Depth of Focus (EDF) patch (NHMW). Photograph of *T.eremitis* male deposited in VMNH were taken with a Canon 9D camera with a 65 mm Canon MP-E macro lens (Canon, Tokyo, Japan) mounted on a Stackshot vertical rail system (Cognisys, Michigan, USA) and focus stacked in Helicon Focus Pro 7 (HeliconSoft, Kharkiv, Ukraine) (VMNH). Drawings of gonopods were executed using a computer monitor and pictures made with a Canon PowerShot A80 digital camera connected to an Axioscope 40 microscope (IZB) or with a DS-Ri-2 camera mounted on a Nikon Eclipse Ni microscope (NHMW). Pictures of specimens were taken using a Nikon DS-Ri-2 camera mounted on a Nikon SMZ25 stereo microscope using NIS-Elements Microscope Imaging Software with an Extended Depth of Focus (EDF) patch (NHMW). For Scanning electron microscopy (SEM) the specimens were: (1) cleaned in an ultrasonic bath (50–60 Hz) for 5 to 10 seconds (maximum), (2) dehydrated in an ascending alcohol series (70%, 80%, 90%, 96% EtOH, 2 × 10–15 min each) and acetone; (3) air dried. Specimens were mounted on aluminum stubs equipped with a sticky aluminum tape, coated with platinum (Leica EM SCD500) and studied with a JEOL JSM 6610-LV at an accelerating voltage of 15 kV or with a JEOL JSM-6460-LV (NHMW). Pictures of live animals were taken with an Olympus Stylus Tough TG-6 (Fig. 2A), Canon PowerShot SX530 HS (Fig. 10A) and a Canon EOS 700D (Fig. 14A) digital camera.

The distribution map was created using Google Earth Pro (ver. 7.3.3.7786) and Adobe Photoshop CS6. The final images were processed with Adobe Photoshop CS6.

### ﻿Gonopod terminology

The description of the basic parts of the gonopods of the new taxa followed Golovatch and VandenSpiegel (2015) with some modifications. The two basic parts of the gonopod are the coxa (cx) with a mesal cannula (ca), and the telopodite. The telopodite is composed of prefemorite (pf) and acropodite (a). The prefemorite is transverse to the main axis of the animal’s body, setose, and makes a nearly right angle with the acropodite. The acropodite is longitudinally divided into two branches, the mesal, solenomeral branch (sb), and the lateral solenophore (sph). Mesally on the prefemorite there is a seminal fossa (sf), from which the seminal groove (sg) starts and runs along the mesal side of the acropodite all the way to the bifurcation point, then passes onto the solenomeral branch and ends with a small opening on the solenomere (s). Detailed and minute structures of the gonopods are explained directly in the figure captions and/or in the text. For more details on the terminology of the Polydesmoidea gonopods, see Shear and Marek (2021).

### ﻿Museum and collection acronyms

**IZB**Institute of Zoology, University of Belgrade – Faculty of Biology, Belgrade, Serbia;

**NHMW**Naturhistorisches Museum Wien, Vienna, Austria;

**NMNHS**National Museum of Natural History, Bulgarian Academy of Sciences, Sofia, Bulgaria;

**VMNH**Virginia Museum of Natural History, Martinsville, Virginia, USA;

**ZMB**Museum für Naturkunde Berlin, Germany;

**ZSM**Zoologische Staatssammlung München, Munich, Germany.

## ﻿Results

### ﻿Class Diplopoda de Blainville in Gervais, 1844

**Order Polydesmida Pocock, 1887**

**Family Trichopolydesmidae Verhoeff, 1910**

#### Taxa from the Carpathians

##### ﻿Genus Bacillidesmus

Taxon classificationAnimaliaPolydesmidaTrichopolydesmidae

Attems, 1898

D2557577-9190-567F-BC0F-6396159A111A

###### Type species.

*Brachydesmusfiliformis* Latzel, 1884, by monotypy.

###### Diagnosis.

**Figure 1.**
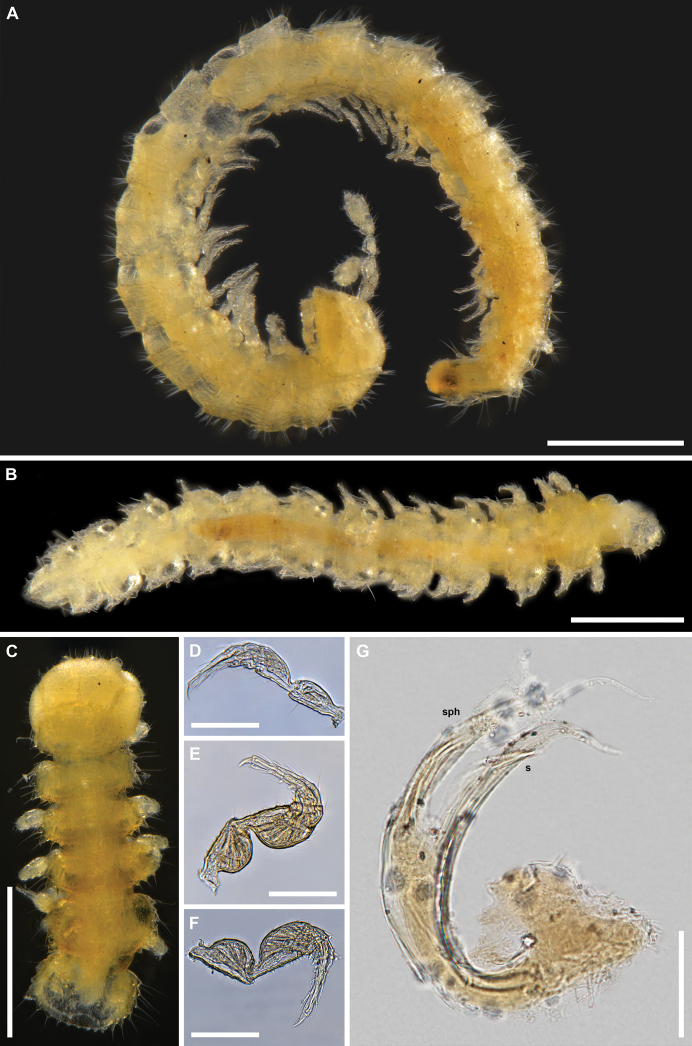
*Bacillidesmusfiliformis* (Latzel, 1884), type material **A** paralectotype ♀ (NHMW MY10266), habitus, lateral view **B** lectotype ♂ (NHMW MY3754) body rings 8–19, dorsal view **C** lectotype ♂ (NHMW MY3754) head and body rings 1–6, dorsal view **D** paralectotype ♀ (NHMW MY10266) left leg 4, posterior view **E** lectotype ♂ (NHMW MY3754) right leg 4, anterior view **F** lectotype ♂ (NHMW MY3754) right leg 10, posterior view **G** lectotype ♂ (NHMW MY3754) gonopod, mesal or lateral view. Abbreviations: **s** solenomere, **sph** solenophore. Scale bars: 0.5 mm (**A–C)**, 0.1 mm (**D–F**), 0.05 mm (**G**).

The monospecific *Bacillidesmus* seems to be the only European Trichopolydesmidae characterized by four regular rows of relatively long trichoid setae on rings 4–18 (Fig. 1A).

In addition, the diagnosis can be amended with the following combination of characters (see also Remarks): small species (4–4.5 mm), 19 body rings (including telson), sensilla basiconica completely enclosed inside the pit of antennomere 6, hypoproct with only two long distal setae, paraprocts with only 2+2 long setae, anterior legs in male with ventral denticles on prefemora, femora, postfemora, tibiae and tarsi (Fig. 1E, F), gonopod telopodite deeply divided into two branches, solenomere (s) and solenophore (sph) situated one below the other, solenomere long and simple, with a lamella, but without additional processes (Figs 1G, 17A–C).

##### ﻿ Bacillidesmus filiformis

Taxon classificationAnimaliaPolydesmidaTrichopolydesmidae

(Latzel, 1884)

08A1EA27-8905-5BAD-97C5-2E44BAB5BE20

Figs 1 , 17A–C

Brachydesmus filiformis Latzel, 1884: 128, 129.
Bacillidesmus filiformis —Attems 1898: 481, figs 97, 98; Attems 1940: 170, fig. 244; Strasser 1962: 443, 444; Strasser 1966a: 341–343; Kime and Enghoff 2011: 72.

###### Diagnosis.

As for the monospecific genus.

###### Material examined.

***Lectotype*** ♂ (NHWM MY3754), designated herewith, “SO Ungarn”, leg. Latzel, don. Latzel 1919. One microslide with only one gonopod. Body in two pieces in ethanol: head with rings 1–6 and rings 8–19; second gonopod, antennae and ring 7 missing.

***Paralectotype*.** 1 ♀ (NHWM MY10266), whole body in ethanol, same data as for lectotype.

###### Distribution.

Unknown.

###### Remarks.

In the original description, Latzel (1884) stated that he analyzed one pair (1 ♂, 1 ♀) that he had collected personally in “südöstlichen Ungarn” (= southeastern part of the Kingdom of Hungary). Later, Strasser (1962) assumed that the species came from “present-day Yugoslavia north of the Danube”. This refers to today’s Vojvodina, northern Serbia. However, the southeastern part of the Kingdom of Hungary included both Banat Mountains and Southern Carpathians (= Transylvanian Alps) in present-day Romania. Bearing in mind that this area is already inhabited by three trichopolydesmid genera, it seems more plausible that *Bacillidesmusfiliformis* could have originated from present-day Romania, rather than northern Serbia which is characterized mainly by agricultural fields. It also remains unknown if this species is cavernicolous or epigean.

This taxon was originally described as *Brachydesmusfiliformis* Latzel, 1884. Attems (1898) analyzed both Latzel’s specimens of *filiformis*, and based on numerous differences with the genus *Brachydesmus* Heller, 1858, he correctly established a new genus, *Bacillidesmus*. At the same time, Attems (1898) gave the first gonopod drawing of this taxon (Fig. 17A). Later, in his famous “Polydesmoidea III”, Attems (1940) provided a new drawing of the *filiformis* gonopod (Fig. 17B), which is slightly different from his 1898 drawing. After studying Attems’ microslide with only one gonopod in poor condition (Figs 1G, 17C) we can confirm that it coincides a bit more with his schematic drawing from 1940. Unfortunately, the second gonopod, as well as ring 7 and both antennae of the lectotype, are most likely lost. It remains unclear whether Attems could have used the now-lost gonopod for the first drawing, or in both cases he used this one, which is still present today, but over time there have been partial changes in its position on the microslide or a partial deformation. Given that Attems (1898) also made a drawing of the antenna, which is missing today, it is very possible that there was another microslide with the second gonopod and antenna/antennae, which we failed to find. However, based on Attems’ (1898, 1940) drawings and the newly examined type material of the gonopod, some conclusions could be drawn here.

The genus *Bacillidesmus* had remained monospecific until Strasser (1962) provisionally included therein a new taxon from Bulgaria, based on a single female. Just a few years later, and this time with males in the hands, Strasser (1966a) confirmed that two more taxa belonged to the genus *Bacillidesmus*, viz., *B.bulgaricusbulgaricus* Strasser, 1962 and *B.bulgaricusdentatus* Strasser, 1966a. However, after a detailed examination of the type material of *Bacillidesmusfiliformis*, as well as material of *B.bulgaricusbulgaricus* and *B.bulgaricusdentatus*, and two related new species from Serbia and Bulgaria, we believe that *Bacillidesmus* should include only *filiformis*, while the remaining aforementioned taxa should be assigned to a new genus, *Balkanodesminus* gen. nov., which we describe below. The new genus differs significantly from *Bacillidesmus* both in somatic and gonopodal characters. The most striking difference in the gonopod structure is that in *Bacillidesmusfiliformis* the solenomeral branch is simple, without a distal solenomeral process, while in *bulgaricusbulgaricus*, *bulgaricusdentatus* and the two new species it is transversely bifid. In addition, these two genera differ significantly in several somatic traits: *Bacillidesmus* has regular rows of metatergal setae, mainly four, whereas *Balkanodesminus* gen. nov. shows 4–8 irregular rows; sensilla basiconica on antennomere 6: completely enclosed inside the pit in *Bacillidesmus*, vs. partially exposed outside the pit in *Balkanodesminus* gen. nov.; setae on paraprocts: 2+2 long setae in *Bacillidesmus*, vs. 2+2 long and ca. 10+10 shorter ones in *Balkanodesminus* gen. nov.; setae on hypoproct: 1+1 long distal setae in *Bacillidesmus*, vs. densely setose, including two long distal setae in *Balkanodesminus* gen. nov.; femora of all male legs swollen in *Bacillidesmus*, vs. only femora of legs 1–3 swollen in *Balkanodesminus* gen. nov.; anterior male legs in *Bacillidesmus* with ventral denticles, vs. denticles absent in *Balkanodesminus* gen. nov. These diferences are sound enough to propose a new genus for the taxa described by Strasser (*B.bulgaricusbulgaricus*, *B.bulgaricusdentatus*) and the two newly described species. Moreover, *Bacillidesmusfiliformis* seems to show more affinity to some of the Carpathian genera (which is another proof that this genus could be from the Carpathians, see under *Banatodesmus* and *Trichopolydesmus*), while *Balkanodesminus* gen. nov., from the Balkan Mountains, shares many similarities with *Rhodopodesmus* gen. nov. (see below).

##### ﻿Genus Banatodesmus

Taxon classificationAnimaliaPolydesmidaTrichopolydesmidae

Tabacaru, 1980

562BDB18-117F-5B91-85EB-98955E24FD7E

###### Type species.

Trichopolydesmus (Banatodesmus) jeanneli Tabacaru, 1980, by monotypy.

###### Diagnosis.

Different from other European Trichopolydesmidae by the presence of an enlarged, oval, paddle-like solenomere (s in Fig. 4), with an additional, small, claw-like, distal solenomeral process (dsp in Fig. 4).

In addition, the diagnosis can be amended with the following combination of characters: medium-sized species (7–7.5 mm), 20 body rings (including telson), rings with 4–6 irregular rows of long trichoid metatergal setae, sensilla basiconica completely enclosed inside the pit of antennomere 6, hypoproct with only two long distal setae, paraprocts with only 2+2 long setae, gonopod acropodite divided into two branches, solenophore (sph in Fig. 4) with three processes, of which the longest is S-shaped (broken off in the SEM image).

##### ﻿ Banatodesmus jeanneli

Taxon classificationAnimaliaPolydesmidaTrichopolydesmidae

(Tabacaru, 1980)

02BA00AB-CD75-5577-98BD-FA5817D764CD

**Figure 2.**
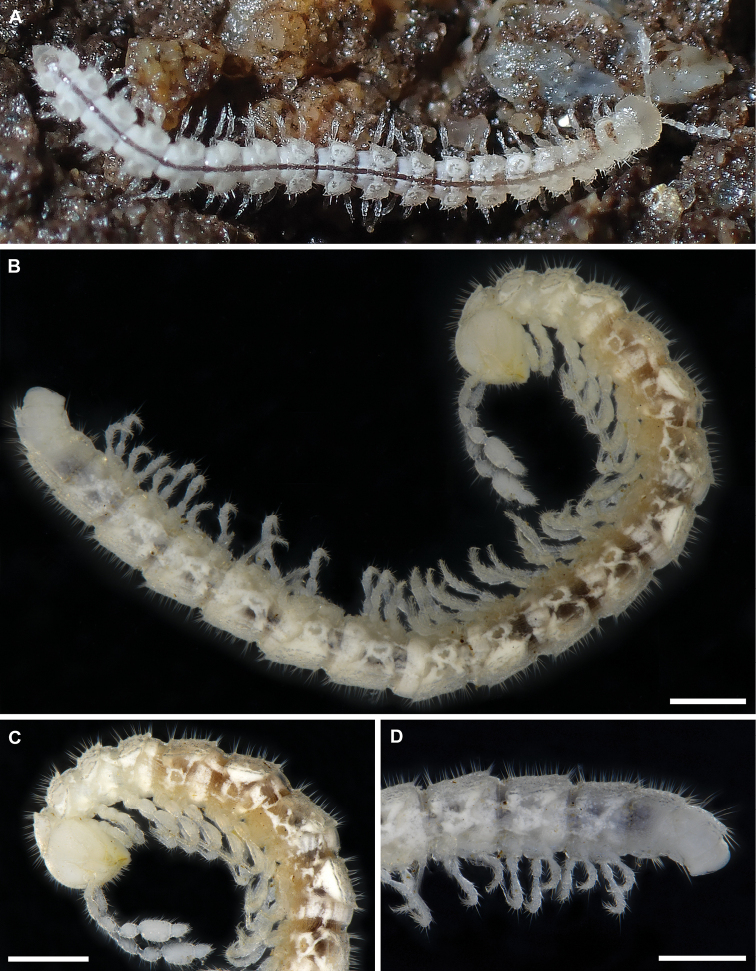
*Banatodesmusjeanneli* (Tabacaru, 1980), ♂(♂) from Peştera Haiducească de la Moldova Nouă Cave, Romania, habitus **A** in situ, dorsal view (photo D. Antić) **B** lateral view (IZB) **C** anterior part of body, lateral view (IZB) **D** posterior part of body, lateral view (IZB). Scale bars: 0.5 mm.

**Figure 3.**
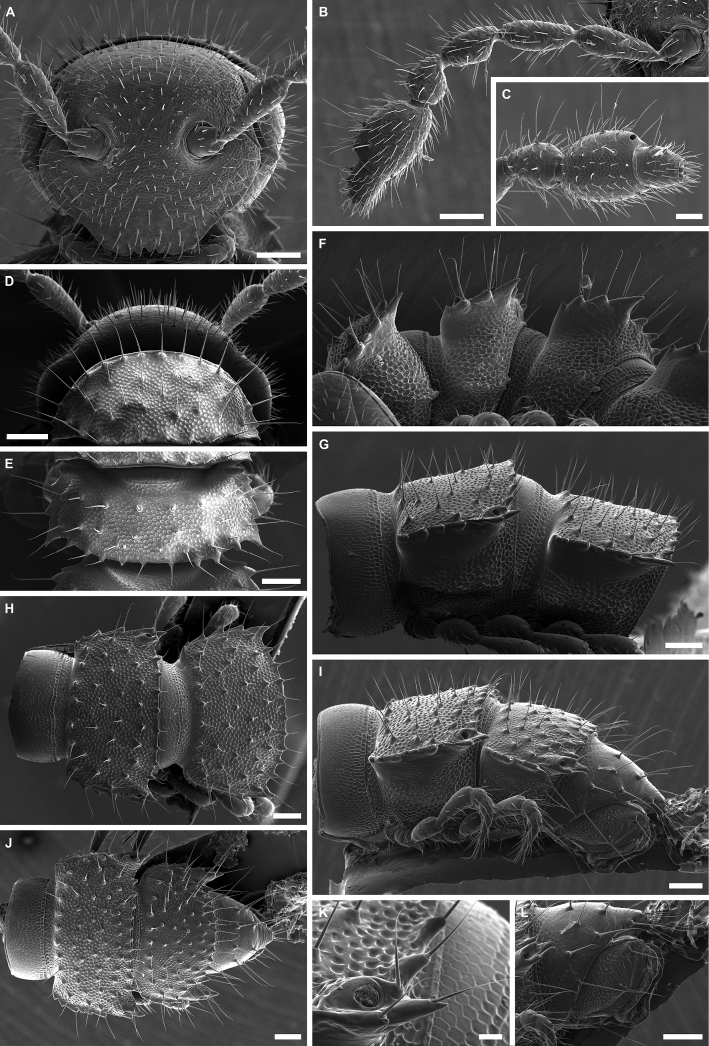
*Banatodesmusjeanneli* (Tabacaru, 1980), ♂ from Peştera Haiducească de la Moldova Nouă Cave, Romania, habitus (NHMW MY10257) **A** head, anterior view **B** right antenna, anterior view **C** distal antennomeres of left antenna, anterior view **D** head and collum, dorsal view **E** body ring 2, dorsal view **F** body rings 2–5, ventro-lateral view **G** body rings 10 and 11, lateral view **H** body rings 10 and 11, dorsal view **I** body rings 18–20, lateral view **J** body rings 18–20, dorsal view **K** left ozopore 10, lateral view **L** telson, lateral view. Scale bars: 0.1 mm (**A, B, D–J, L**), 0.05 mm (**C**), 0.02 mm (**K**).

**Figure 4.**
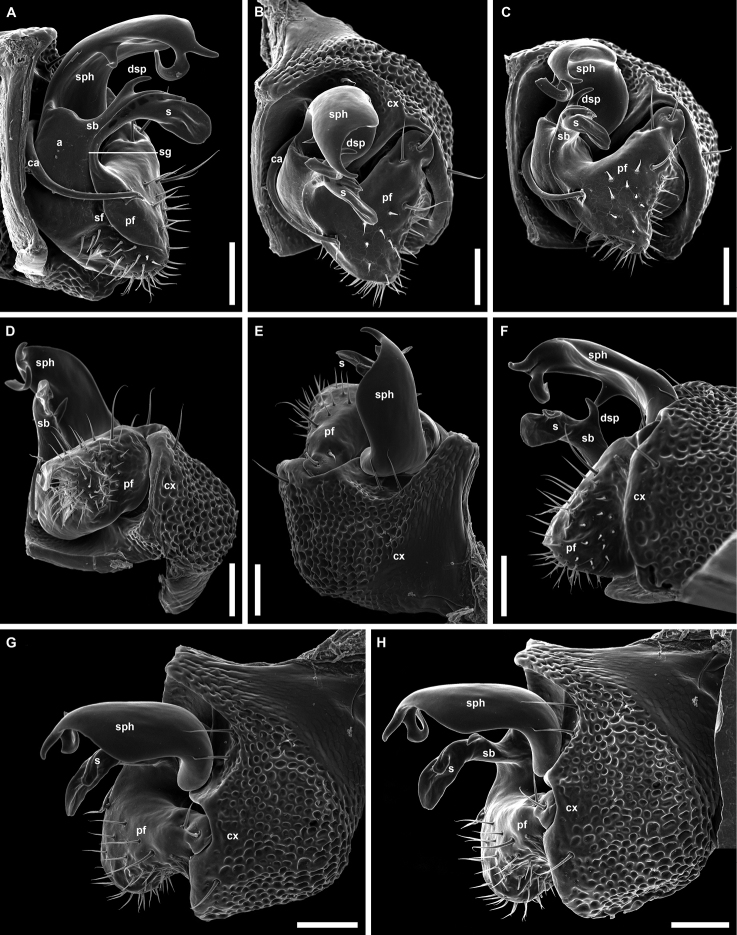
*Banatodesmusjeanneli* (Tabacaru, 1980), ♂ from Peştera Haiducească de la Moldova Nouă Cave, Romania, left gonopod (NHMW MY10257) **A** mesal view **B** antero-distal view **C** distal view **D** posterior view **E** anterior view **F** lateral view **G, H** antero-lateral views. Abbreviations: **a** acropodite, **ca** cannula, **cx** coxa, **dsp** distal solenomeral process, **pf** prefemorite, **s** solenomere, **sb** solenomeral branch, **sf** seminal fossa, **sg** seminal groove, **sph** solenophore. Tip of “S” shaped process of **sph** broken. Scale bars: 0.05 mm.

Figs 2 , 3 , 4 , 17D , 18

Trichopolydesmus (Banatodesmus) jeanneli Tabacaru, 1980: 156, figs 1–3.
Trichopolydesmus jeanneli —Ceuca 1992: 416.
Trichopolydesmus (Banatodesmus) jeanneli —Giurginca 2021: 86, fig. 52 (Banatodesmus obviously mistakenly listed as a subgenus, see below).
Banatodesmus jeanneli —Tabacaru 1996: 68, fig. 1A; Tabacaru et al. 2003: 133; Tabacaru and Giurginca 2016: 101, fig. 14C, D; Kime and Enghoff 2011: 72, 262.

###### Diagnosis.

As for the monospecific genus.

###### Material examined.

1 ♂, 1 ♀ (IZB), Romania, Banat, Moldova Noua, Peştera Haiducească de la Moldova Nouă Cave (= Gaura Turceasca, Grota Haiducilor), 44.7314, 21.7394, 28.X.2021, leg. D. Antić & D. Stojanović, 1 ♂ (used for SEM, NHMW MY10257), same data as for the previous material.

###### Distribution.

This species has been described and is still known only from two caves in the Banat Mountains in Romania, Peştera Haiducească de la Moldova Nouă and Peştera de la Lacul Dracului caves (Fig. 18).

###### Remarks.

Originally, *Banatodesmus* was described as a subgenus of *Trichopolydesmus* Verhoeff, 1898 (Tabacaru 1980). Later, Mauriès (1984) reasonably considered it as a separate genus, this being generally accepted today. Recently, in his book on Romanian millipedes, Giurginca (2021) referred to it as “Trichopolydesmus (Banatodesmus) jeanneli”. This was apparently a mistake, since in the rest of the text *Banatodesmus* was clearly treated as a genus.

The sample examined here is the first record of this taxon since its original description. Two males and one female were discovered at one of the two type localities, Peştera Haiducească de la Moldova Nouă Cave. It is interesting that all three specimens were collected not far from the entrance to the cave, within one square meter, near a small stream that flows through the cave. The female was found under a piece of rotten wood, while both males were taken from under two deeply embedded stones.

Although Tabacaru (1980) provided an excellent description and very fine drawings (Fig. 17D) of this taxon, the recently found specimens gave us the opportunity to document this taxon with photographs and SEM images of the habitus and gonopods (Figs 2–4).

As mentioned above, *Bacillidesmusfiliformis* seems to show some habitual and gonopodal similarities with *Banatodesmus*. Both taxa share sensilla basiconica of antennomere 6 completely enclosed in the pit, paraprocts with only two long setae each, and hypoproct with only two long distal setae. In addition, the solenomeral branch and the solenophore are oriented mostly antero-posteriorly rather than meso-laterally to each other.

##### ﻿Genus Napocodesmus

Taxon classificationAnimaliaPolydesmidaTrichopolydesmidae

Ceuca, 1974

247550FE-1466-565E-842F-3E0975CC6551

###### Type species.

*Napocodesmusendogeus* Ceuca, 1974, by monotypy.

###### Diagnosis.

This is the only European genus of Trichopolydesmidae that is characterized by hook-shaped posterolateral cones on metaterga, see Ceuca (1974) and Tabacaru (1975).

##### ﻿ Napocodesmus florentzae

Taxon classificationAnimaliaPolydesmidaTrichopolydesmidae

Tabacaru, 1975

313CB9B6-5A38-5E1C-8A60-599600B74BCA

Figs 17E, F , 18

Napocodesmus florentzae Tabacaru, 1975: 73, figs 1–6.
Napocodesmus florentzae —Ceuca 1992: 416; Tabacaru et al. 2003: 133; Tabacaru and Giurginca 2016: 100, fig. 13; Kime and Enghoff 2011: 72, 265; Giurginca 2021: 88, fig. 54.

###### Diagnosis.

Cannot be compared to *N.endogeus* since its description was based on females only (see under Remarks).

Besides the hook-shaped posterolateral cones on the metaterga, this species differs from other European Trichopolydesmidae by the simplified gonopods with the acropodite divided in its distal third into two branches, a slender and claw-like solenophore and a wide and flattened, sublamelliform solenomere, both branches being parallel and oriented completely meso-laterally to each other (Fig. 17E, F).

In addition, the diagnosis can be amended with the following combination of characters: small species (3.4 mm), 19 body rings (including telson), sensilla basiconica on antennomere 6 partially exposed outside the pit, hypoproct with more than two long distal setae, paraprocts with more than 2+2 long setae, metaterga with 4–7 irregular rows of trichoid setae.

###### Distribution.

This species is known only from its type locality, Peştera cu Două Uşi Cave, Sușița Verde Valley, Vâlcan Mountains, Gorj County, Romania (Fig. 18).

###### Remarks.

Tabacaru (1975) stated that he had collected a male and a female, but that the female was lost during a breeding experiment. The excellent description and drawings (Fig. 17E, F) he gave were based on only one male, which, if it still exists, should be treated as the holotype by monotypy.

The type species of this genus, *N.endogeus*, was described based on nine females found in the soil near the Biology Department at the University of Cluj in Romania (Ceuca 1974). Akkari and Enghoff (2011) cited this species from deep soil in an orchard in Moldova. Before that, Golovatch and Kime (2009) stated that this species is very common and abundant in Moldova’s apple orchards, but probably accidentally under the name *N.florentzae*, instead of *N.endogeus*.

*Napocodesmusflorentzae* shares some similarities in its habitus with *Balkanodesminus* gen. nov. and *Rhodopodesmus* gen. nov., viz., small size, 19 body rings, 4–7 rows of irregular trichoid setae (4–8 in two last-mentioned genera), sensilla basiconica on antennomere 6 partially exposed outside the pit, and hypoproct and paraprocts with more than two long setae.

##### ﻿Genus Trichopolydesmus

Taxon classificationAnimaliaPolydesmidaTrichopolydesmidae

Verhoeff, 1898

E1BB136A-3CCC-52EA-9F6B-768B9CDEB471

###### Type species.

*Trichopolydesmuseremitis* Verhoeff, 1898, by monotypy.

###### Diagnosis.

Differs from other European Trichopolydesmidae by the gonopod acropodite divided into three branches, where solenomere is thin, long and acuminate, and devoid of additional process.

**Figure 5.**
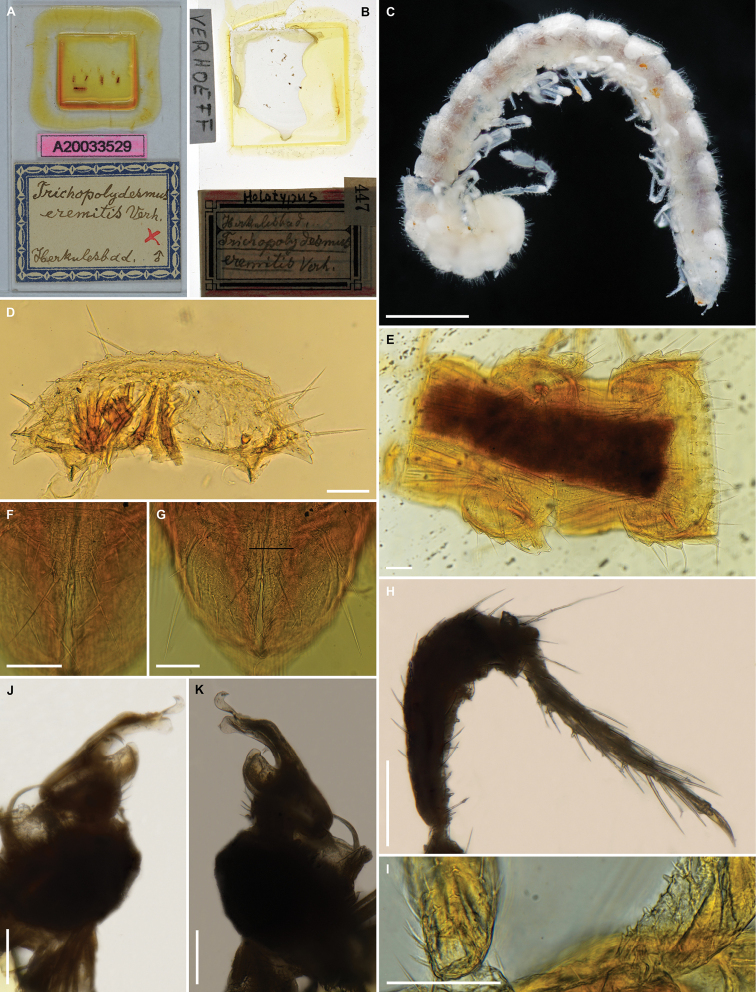
*Trichopolydesmuseremitis* Verhoeff, 1898 **A, D–F, I** holotype ♂ (ZSM-A20033529) **B, H, J, K** holotype ♂ (ZMB 13160) **C** ♂ (VMNH110683) **A** microslide with habitus parts **B** microslide with gonopods and one lag pair **C** habitus, lateral view **D** collum, dorsal view **E** body rings 13 and 14, dorsal view **F** hypoproct, ventral view **G** telson, ventral view **H** leg ?7 **I** left legs 10 and 11 **J, K** ?left gonopod, distomesal and distoanterolateral views, respectively. Scale bars: 0.5 mm (**C**), 0.1 mm (**D–K**).

In addition, the diagnosis can be amended with the following combination of characters: medium-sized species (8.5 mm), 20 body rings (including telson), sensilla basiconica on antennomere 6 partially exposed outside the pit, paraprocts and hypoproct densely setose (Fig. 5F, G), metaterga with 4–6 irregular rows of long trichoid setae, podomeres of anterior legs in males with denticles on their ventral side, tarsi with rare sphaerotrichomes (Fig. 5H, I).

##### ﻿ Trichopolydesmus eremitis

Taxon classificationAnimaliaPolydesmidaTrichopolydesmidae

Verhoeff, 1898

1DAEE905-9A0C-5E38-A808-390EEEA35729

Figs 5 , 17G, H , 18

Trichopolydesmus eremitis Verhoeff 1898: 363, figs 6–8.
Trichopolydesmus eremitis —Attems 1899: 429; Attems 1940: 168, fig. 240; Verhoeff 1941a: 186, figs 15, 16; Verhoeff 1941b: 44, figs 47, 48; Ceuca 1958: 340, figs 7–9; Ceuca 1992: 416; Tabacaru et al. 2003: 133; Tabacaru and Giurginca 2016: 100, fig. 14A, B; Kime and Enghoff 2011: 72, 266; Giurginca 2021: 89, fig. 55.

###### Diagnosis.

As for the monospecific genus.

###### Material examined.

***Holotype*** ♂ (by monotypy, two microslides: ZSM-A20033529 and ZMB 13160), Herkulesbad (Băile Herculane, Romania), leg. K. Verhoeff. ZSM-A20033529 (Fig. 5A): head in several pieces, only first three antennomeres of one antenna, collum, rings 3–5, 8–10, 12, 13–14, 15–20. ZMB 13160 (Fig. 5B): gonopods, one leg pair (7?).

###### Additional material.

1 ♂ (VMNH110683), body in two pieces in alcohol (Fig. 5C), ring 7 and gonopods missing. For more details see below.

###### Distribution.

Known from several caves in the southern part of the Carpathians in Romania: Peştera Hoţilor de la Băile Herculane (type locality), Peştera nr. 40 de la Ineleţ, Peştera Cicioara, Peştera Cornetul Vârcanilor, Peştera Cloşani, Peştera Vacilor de la Cloşani and Peştera din Poiana Lazului (= Peştera lui Mihai Arjoc, = Peştera din Piatra Mică) (Verhoeff 1898; Ceuca 1958; Tabacaru et al. 2003) (Fig. 18).

###### Remarks.

Verhoeff (1898) described this taxon from a single male he collected in the Hoţilor Cave in Băile Herculane. As he himself stated, several subsequent attempts to collect additional specimens in this cave were unsuccessful. Tabacaru (1980) stated that numerous searches in this cave failed too. One of us (DA) visited this cave in 2014 but also failed to find this species. In 2021, a small group of myriapodologists, including two of us (DA and BV) were not successful either. From Hoţilor Cave, only the male type specimen originally described by Verhoeff (1898) is known.

Sixty years after its original description, Ceuca (1958) examined more than 20 specimens of *T.eremitis* from three other caves and gave new and more detailed drawings of the gonopods (Fig. 17G, H), as well as some notes on female habitus.

Hoffman (1980) wrote that he had received a male from Traian Ceuca, whose photograph is included in this paper (Fig. 5C). Unfortunately, colleague Jackson Means informed us that there is no original label with this individual, but that on the jar, marked with MIR02733, it is written: “Trichopolydesmidae: *Trichopolydesmuseremitis* Verhoeff TOPOTYPES !! Hungary”. This was probably an accidental mistake during the subsequent labeling. The male sent by Ceuca to Hoffman comes from one of the three caves in Romania listed in Ceuca’s (1958) paper. Considering the number of collected males from those three caves, we can only guess that this male comes from the Cloşani cave.

Similarly to *Bacillidesmusfiliformis*, this species also has ventral denticles on podomeres of male anterior legs (Fig. 5H, I). However, some other habitual characteristics are similar to *Napocodesmus*, *Balkanodesminus* gen. nov. and *Rhodopodesmus* gen. nov., viz., sensilla basiconica on antennomere 6 partially exposed outside the pit, while hypoproct and paraprocts are with more than two long setae (Fig. 5F, G). Legs and antennomeres (as well as antennae in general) in this species are somewhat longer (slender) than in other representatives from the Carpathian-Balkan arch and the Rhodope Mountains, thus it seems to be the most strongly adapted to cave life among them.

#### Taxa from the Balkan (Stara Planina) Mountain range

(Besides Stara Planina Mountain, this range includes the Predbalkan in Bulgaria, as well as numerous mountains in eastern Serbia)

##### ﻿Genus Balkanodesminus gen. nov.

Taxon classificationAnimaliaPolydesmidaTrichopolydesmidae

8547ED60-9E0F-597D-B018-5D40747E9947

http://zoobank.org/B53F3D76-3549-4DD0-96BC-8BD848613381

###### Type species.

*Bacillidesmusbulgaricus* Strasser, 1962, by present designation.

###### Diagnosis.

Differs from all European Trichopolydesmidae by the presence of a characteristic acropodite of the gonopods divided into two parallel and mostly meso-laterally oriented branches, where solenomeral branch is transversely bipartite, consisting of slender solenomere and well-developed distal solenomeral process. The most similar genus is *Rhodopodesmus* gen. nov., but it differs from *Balkanodesminus* gen. nov. and all other European Trichopolydesmidae by the presence of trifid solenomeral branch (for more details see under *Rhodopodesmus* gen. nov.).

In addition, the diagnosis can be amended with the following combination of characters: small size (3.7–5.2 mm), 19 body rings (including telson), sensilla basiconica on antennomere 6 partially exposed outside the pit, hypoproct with more than two long distal setae, paraprocts with more than 2+2 long setae, metaterga with 4–8 irregular rows of trichoid setae.

###### Name.

The new genus is named after the Balkan Mountains, its type locality, in combination with the suffix -*desminus*, as a diminutive of -*desmus*, the common suffix in Polydesmida, referring to the small size of its species, in contrast to confamiliar Dinaric *Balkanodesmus* Antić & Reip, in Antić et al. 2014, the largest Balkan trichopolydesmid. The name is a masculine noun.

###### Included species.

*Balkanodesminusbulgaricus* (Strasser, 1962) gen. nov., comb. nov. ex *Bacillidesmus*

*Balkanodesminusdentatus* (Strasser, 1966a) gen. nov., comb. nov., stat. nov. ex *Bacillidesmus*

*Balkanodesminusdentatoides* gen. nov. et sp. nov.

*Balkanodesminusserbicus* gen. nov. et sp. nov.

##### ﻿ Balkanodesminus bulgaricus

Taxon classificationAnimaliaPolydesmidaTrichopolydesmidae

(Strasser, 1962) gen. nov., comb. nov. ex Bacillidesmus

D6426609-EF94-5B1E-B52A-C6D2B943481A

Figs 6 , 17I , 18

Bacillidesmus? bulgaricus Strasser, 1962: 443, figs 7–10.
Bacillidesmus bulgaricus bulgaricus —Strasser 1966a: 341, figs 13–15; Strasser 1973: 419; Stoev 2004: 149; Stoev 2007: 384; Beron 2015: 80, 410; Bachvarova et al. 2017: 521.
Bacillidesmus bulgaricus —Ceuca 1992, 416; Kime and Enghoff 2011: 71, 262.

###### Diagnosis.

Differs from *Balkanodesminusdentatoides* gen. nov. et sp. nov. and *B.dentatus* gen. nov., comb. nov., stat. nov. by the presence of longer (vs. shorter) metatergal setae and their smaller (vs. greater) number of rows, as well as by the presence of more simplified gonopods, with uniramous (vs. biramous) distal solenomeral process, and smaller and smooth (vs. larger and denticulated) lamella of solenophore. From *B.serbicus* gen. nov. et sp. nov., with which it shares similar habitus and similar gonopods, it differs by the presence of larger (vs. smaller) lamella of solenophore, slender, almost straight (vs. more robust and sigmoid) distal projection of solenophore, distal projection without (vs. with) basal lobe and slenderer (vs. stouter) solenomere and distal solenomeral process, where the solenomere exceeds the distal solenomeral process by ½ (vs. ¼) of its length.

**Figure 6.**
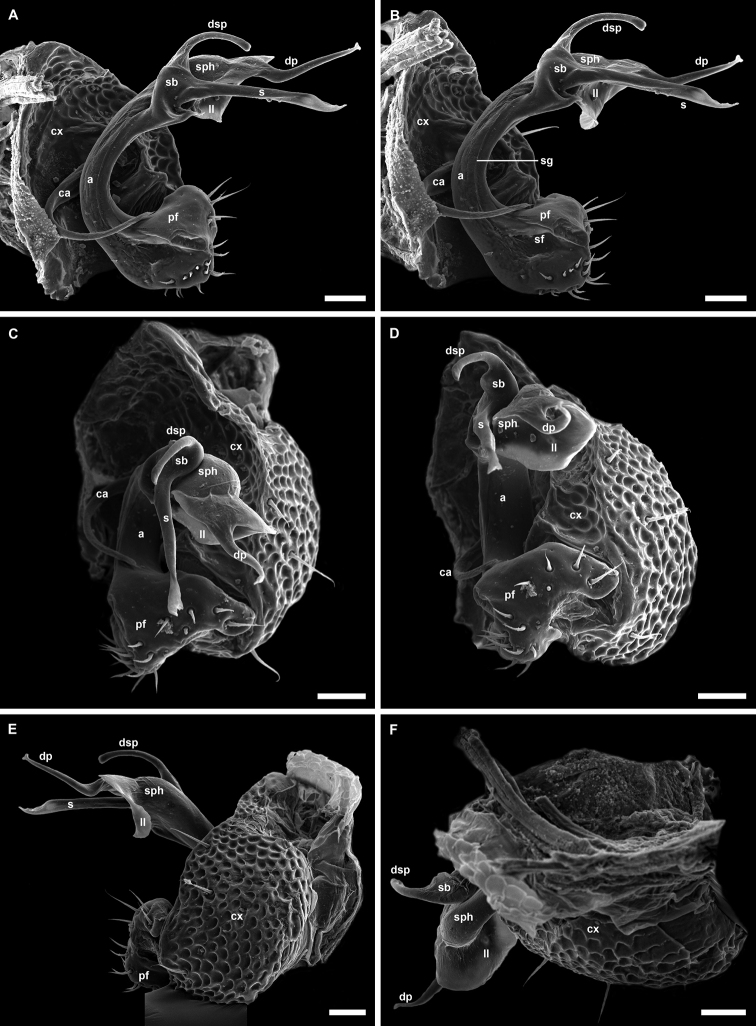
*Balkanodesminusbulgaricus* (Strasser, 1962) gen. nov., comb. nov., ♂ from Ponora Cave, Bulgaria, left gonopod (NMNHS-10813) **A, B** mesal views **C** antero-distal view **D** distal view **E** lateral view **F** anterior view. Abbreviations: **a** acropodite, **ca** cannula, **cx** coxa, **dp** distal projection of solenophore, **dsp** distal solenomeral process, **ll** lamella of solenophore, **pf** prefemorite, **s** solenomere, **sb** solenomeral branch, **sf** seminal fossa, **sg** seminal groove, **sph** solenophore. Scale bars: 0.02 mm.

###### Material examined.

2 ♂♂, 6 ♀♀ (NMNHS-10813), Bulgaria, Vratsa District, Chiren, Ponora Cave, clay, 27.I.1998, leg. B. Petrov & T. Ivanova.

###### Distribution.

This species shows a somewhat scattered distribution (see Fig. 18). It is present in caves starting from Vidin Municipality, through Chuprene and Chiprovtsi municipalities all the way to Vratsa municipality. These are Varkan Cave, Vidin Municipality (Strasser 1973), Desni suhi pech Cave, Chuprene Municipality (Strasser 1973), Mishin kamik Cave (type locality) and Vreloto v seloto Cave, both Chiprovtsi Municipality (Strasser 1962, 1966a), as well as Mladenovata peshtera Cave and Ponora Cave, Vratsa Municipality (Strasser 1966a; Stoev 2004).

###### Remarks.

Strasser (1962) described this species based on a poorly preserved female that he placed with uncertainty in the genus *Bacillidesmus*. He emphasized a very important difference in sensilla basiconica on 6^th^ antennomere being partially exposed in *bulgaricus*, while they are completely enclosed in their pit in *filiformis*.

Four years later, when males became available, Strasser (1966a) confirmed that *bulgaricus* belonged to the genus *Bacillidesmus*, based on some similarities in gonopod structures, and identified two subspecies, viz., *B.bulgaricusbulgaricus* and *B.bulgaricusdentatus*. Subsequently, both subspecies were recognized as such by Stoev (2004, 2007), Beron (2015) and Bachvarova et al. (2017). On the other hand, Ceuca (1992), and Kime and Enghoff (2011) considered *dentatus* as a separate species, *B.dentatus*. Here, we treat both taxa as separate species (see below).

Based on the distribution of the genus *Balkanodesminus* gen. nov., and the scattered distribution of *B.bulgaricus* gen. nov., comb. nov., we are not excluding the possibility that not all records of *bulgaricus* are in fact of that species. Illustrations of gonopods are known only from the two easternmost populations, from the Mladenovata peshtera Cave (Strasser 1966a) and Ponora Cave (present study).

##### ﻿ Balkanodesminus dentatus

Taxon classificationAnimaliaPolydesmidaTrichopolydesmidae

(Strasser, 1966a) gen. nov., comb. nov. stat. nov.

F4E1B903-9575-5DA2-A391-C0CE651C5942

Figs 17L , 18

Bacillidesmus bulgaricus dentatus Strasser, 1966a: 341, figs 16, 17.
Bacillidesmus bulgaricus dentatus in part.—Stoev 2007: 384; Beron 2015: 80, 411; Bachvarova et al. 2017: 521.
Bacillidesmus dentatus in part.—Ceuca 1992: 416; Kime and Enghoff 2011: 71, 262.
**not**Bacillidesmusbulgaricusdentatus —Stoev 2004: 149.

###### Diagnosis.

Differs from *Balkanodesminusbulgaricus* gen. nov., comb. nov. and *B.serbicus* gen. nov. et sp. nov. by the presence of shorter (vs. longer) metatergal setae and their greater (vs. smaller) number of rows, as well as by the presence of more complicated (vs. more simplified) gonopods, with biramous (vs. uniramous) distal solenomeral process and larger and denticulated (vs. smaller and smooth) lamella of solenophore. From *B.dentatoides* gen. nov. et sp. nov., with which it shares similar habitus and similar gonopods, it differs by the presence of more robust (vs. slenderer) solenomere, by short process (vs. triangular tooth) on distal solenomeral process, and by the presence (vs. absence) of additional short subdistal process at distal projection of solenophore. In addition, lateral lamella and basal lobe less developed than in *B.dentatoides* gen. nov. et sp. nov.

###### Material examined.

1 ♀, 1 juv. (NMNHS-10814), Bulgaria, Vratsa District, Byala Slatina Municipality, Drashan, Drashanskata peshtera Cave (type locality), 22.IX.1992, leg. P. Beron.

###### Distribution.

So far, this species is known only from its type locality, Drashanskata peshtera Cave (Fig. 18).

###### Remarks.

Originally described as a subspecies, *Bacillidesmusbulgaricusdentatus*. Strasser (1966a) pointed out significant differences in the structure of the gonopod between *bulgaricus* and *dentatus*, as well as differences in body size. However, he did not notice the differences in the length and arrangement of metatergal setae between the two taxa. The descriptions of the two new species below, one of which is similar to *bulgaricus* in body size, habitus and gonopods, and the other one to *dentatus*, clearly indicate the presence of two groups of species within this genus.

As mentioned above, Ceuca (1992), and Kime and Enghoff (2011) treated this taxon as a separate species, *Bacillidesmusdentatus*, without, however, any formal taxonomic act. For the sake of stability of nomenclature here we formally raise this taxon to the species level and transfer it to the newly established genus as *Balkanodesminusdentatus* gen. nov., comb. nov., stat. nov.

##### ﻿ Balkanodesminus dentatoides

Taxon classificationAnimaliaPolydesmidaTrichopolydesmidae

gen. nov. et sp. nov.

DEA33465-20AD-5089-A533-0148CDBDED5E

http://zoobank.org/BEF9EB03-1DC8-4C13-BE0F-DE8A2AD0C257

**Figure 7.**
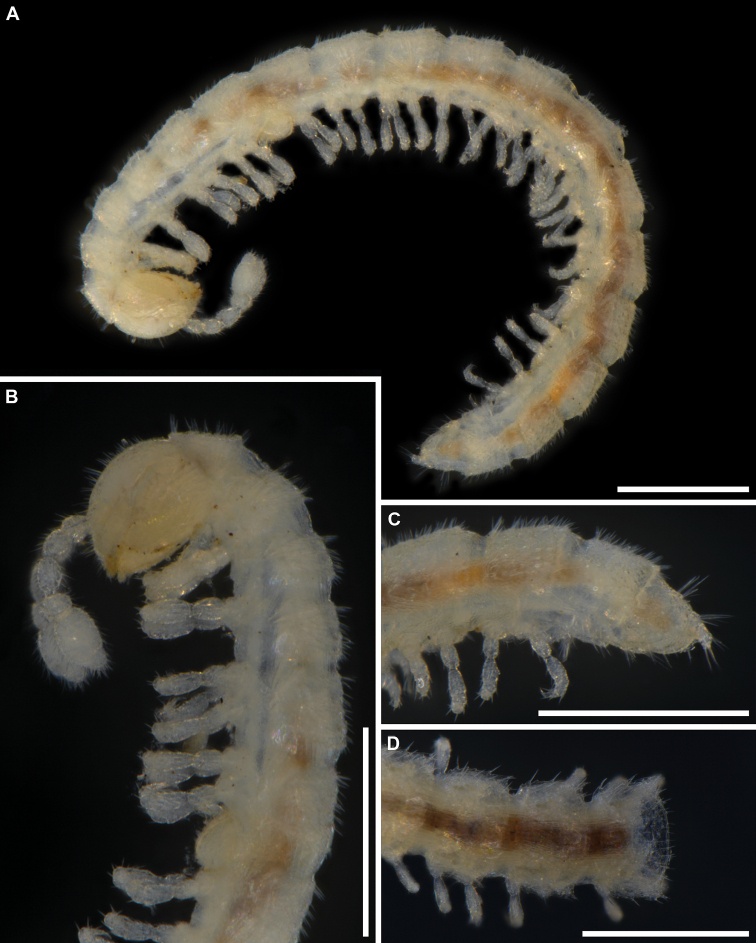
*Balkanodesminusdentatoides* gen. nov. et sp. nov., holotype ♂ (**A–C**, NMNHS-10815) and paratype ♂ (**D**, NHMW MY10258), habitus **A** lateral view **B** anterior part of body, lateral view **C** posterior part of body, lateral view **D** midbody rings, dorsal view. Scale bars: 0.5 mm.

**Figure 8.**
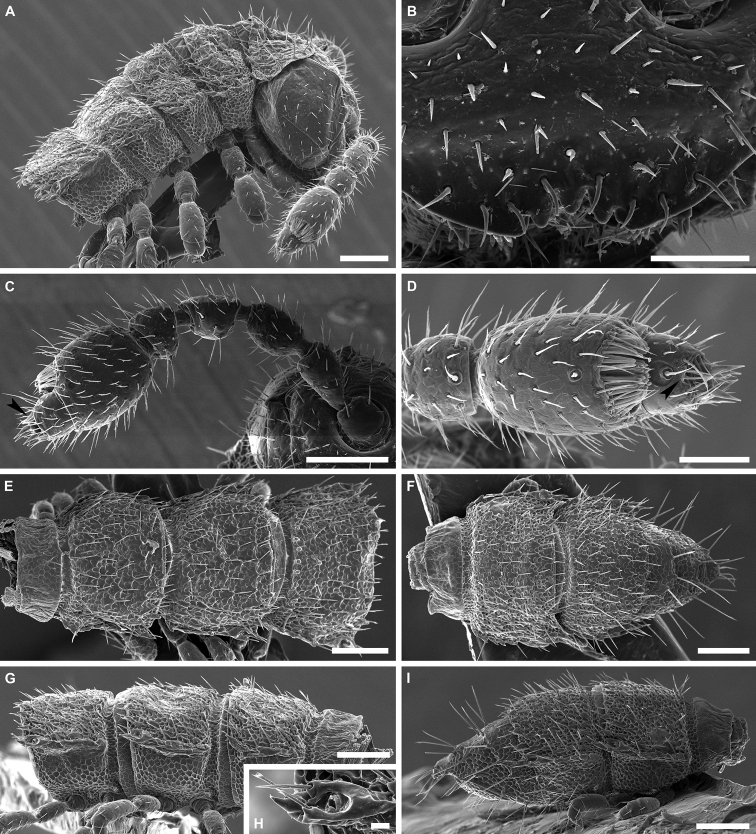
*Balkanodesminusdentatoides* gen. nov. et sp. nov., paratype ♂ habitus (NHMW MY10258) **A** anterior part of body, lateral view **B** labrum, anterior view **C** right antenna, anterior view (arrow indicates small bulge with sensilla basiconica spiniformia) **D** distal antennomeres of right antenna, dorsal view (arrow indicates small bulge with sensilla basiconica spiniformia) **E, F** body rings 8–10, lateral and dorsal views, respectively **G** right ozopore 10, lateral view **H, I** body rings 17–19, dorsal and lateral views, respectively. Scale bars: 0.1 mm (**A, C, E, F, H, I**), 0.05 mm (**B, D**), 0.01 mm (**G**).

**Figure 9.**
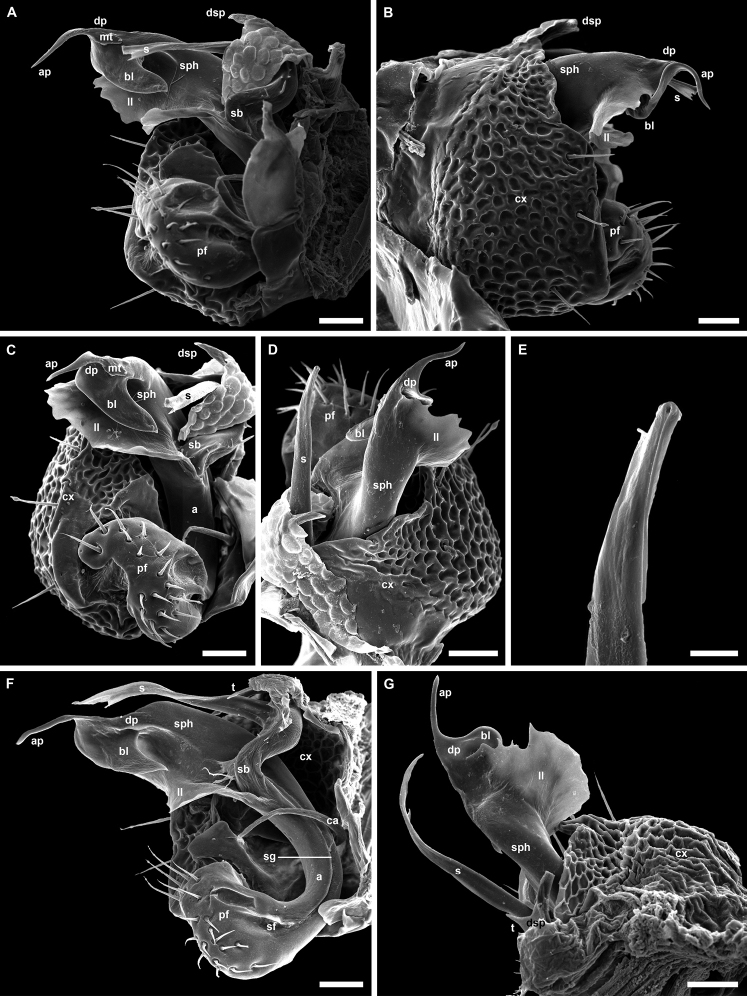
*Balkanodesminusdentatoides* gen. nov. et sp. nov., right gonopods **A–E** paratype ♂ (NHMW MY10258) **A** mesal view **B** lateral view **C** meso-distal view **D** antero-distal view **E** tip of solenomere, distal view **F, G** ♂ from Sopotska peshtera Cave (NHMW MY10267), mesal and antero-disto-lateral views, respectively. Abbreviations: **a** acropodite, **ap** acuminate process of solenophore, **bl** basal lamellar lobe of solenophore, **ca** cannula, **cx** coxa, **dp** distal projection of solenophore, **dsp** distal solenomeral process, **ll** lamella of solenophore, **mt** mesal thickening of solenophore, **pf** prefemorite, **s** solenomere, **sb** solenomeral branch, **sf** seminal fossa, **sg** seminal groove, **sph** solenophore, **t** mesal tooth of distal solenomeral process. Scale bars: 0.02 mm (**A–D, F, G**), 0.005 mm (**E**).

Figs 7 , 8 , 9 , 17M, N , 18

Bacillidesmus bulgaricus dentatus —Stoev 2004: 149.
Bacillidesmus bulgaricus dentatus in part.—Stoev 2007: 384; Beron 2015: 80, 411; Bachvarova et al. 2017: 521; Kime and Enghoff 2011: 71, 262.

###### Diagnosis.

Differs from *Balkanodesminusbulgaricus* gen. nov., comb. nov. and *B.serbicus* gen. nov. et sp. nov. by the presence of shorter (vs. longer) metatergal setae and their greater (vs. smaller) number of rows, as well as by the presence of more complicated (vs. more simplified) gonopods, with biramous (vs. uniramous) distal solenomeral process and larger and denticulated (vs. smaller and smooth) lamella of solenophore. From *B.dentatus* gen. nov., comb. nov., stat. nov., with which it shares similar habitus and similar gonopods, it differs by the presence of slenderer (vs. more robust) solenomere, by small triangular tooth (vs. short process) on distal solenomeral process, and by the absence (vs. presence) of additional short subdistal process at distal projection of solenophore. In addition, lateral lamella and basal lobe more robust than in *B.dentatus* gen. nov., comb. nov., stat. nov.

###### Name.

The species name is a combination of the name *dentatus* and the Ancient Greek suffix -*oides*, referring to the species’ particularly strong resemblance to *Balkanodesminusdentatus* gen. nov., comb. nov., stat. nov. Adjective.

###### Material examined.

***Holotype*** ♂ (NMNHS-10815), Bulgaria, Lovech District, Yablanitsa Municipality, Brestnitsa, Saeva dupka Cave, clay, 13.X.1997, leg. B. Petrov & P. Stoev.

***Paratypes*.** 2 ♂♂ (NMNHS-10816, 10817), 1 ♀, (NMNHS-10818), same data as for holotype; 1 ♂ (used for SEM, NHMW MY10258) same data as for holotype.

###### Additional material.

1 ♂ (right gonopod used for SEM, NHMW MY10267), 2 ♂♂ (NMNHS-10819, 10820), 2 ♀♀ (NMNHS-10821, 10822), Lovech District, village of Sopot, Sopotska peshtera Cave, 8.V.2004, leg. P. Beron.

###### Description.

***Number of body rings and measurements***: Body with 19 rings (including telson) in adults, moniliform. Holotype male 3.8 mm long, width of midbody pro- and metazonae 0.25 mm and 0.30 mm, respectively. Paratype males 3.4–3.9 mm long, width of midbody pro- and metazonae 0.23–0.25 mm and 0.29–0.31 mm, respectively. Paratype female 4.3 mm long, width of midbody pro- and metazona 0.30 and 0.35 mm, respectively.

***Coloration***: Entirely pallid, slightly translucent (Fig. 7).

***Head***: Broader than collum, setose (Fig. 7B); epicranial suture poorly developed; isthmus between antennae ≈ 1.3 × broader than diameter of antennal socket. Labrum with three labral teeth, and with 3+3 labral and five supralabral setae (Fig. 8B). Gnathochilarium without peculiarities. Antennae rather short, clavate (Figs 7B, 8C, D). Antennomere length 6 > 2 = 3 = 4 > 5 > 7 = 1. Antennae 0.6 mm long in the holotype male; length/breadth ratios of antennomeres 1–7: 1 (1), 2 (2), 3 (2), 4 (2), 5 (1), 6 (1) and 7 (1). Antennomere 6 with four sensilla trichodea and with strongly developed disto-dorsal pit with numerous long sensilla basiconica partially exposed outside the pit (Fig. 8D). Antennomere 7 with one sensillum trichodeum and a small bulge with three sensilla basiconica spiniformia (Fig. 8C, D). Four apical cones (Fig. 8D).

***Collum***: Semi-circular, with one or two lateral incisions and ≈ 7 irregular rows of medium sized and trichoid setae.

***Body rings***: Tegument shining, texture alveolate, reticulate and scaly. Rings densely setose. Setae rather short and trichoid, originating from small tubercules (Figs 7, 8A, E–G, I). Posteriormost tubercules mostly with a small thorn. Rings 2–4 with ≈ 4 rows of setae (Fig. 8A). Rings 5–18 with ≈ 6–8 irregular rows of setae (Fig. 8E–G, I). Paraterga serrated, with 5–7 teeth (Figs 7D, 8E–G, I). Pore formula normal: 5, 7, 9, 10, 12, 13, 15–18. Poriferous metazonae with an enlarged postero-lateral cone bearing an ozopore and three medium-sized setae (Fig. 8H). Epiproct blunt, directed slightly ventrad (Figs 7C, 8F, I). Paraprocts semi-spherical, each with two long setae originating from small tubercules and ≈ 10 shorter setae without tubercules (Fig. 8I). Hypoproct trapeziform with 2 long distal setae and numerous shorter setae throughout (Fig. 7C). Sterna unmodified, poorly setose. Pleurosternal carinae absent, only a few small teeth sometimes present on rings 2 and 3 (Fig. 8A). Gonopod aperture large, subsemi-circular.

***Walking legs***: Legs 1–3 in males with swollen femur; coxa 2 with a short mesal apophysis (cf. Strasser 1966a: 341, fig. 13). No other peculiarities.

***Gonopods*** (Figs 9, 17M, N): Coxa (cx) large, semi-circular in ventral and lateral views, with differentiated gonocoel mesally; lateral part swollen, alveolate, with three long setae near mesal ridge. Cannula (ca) long, C-shaped. Telopodite relatively long compared to coxa, consisting of a transverse, setose prefemorite (pf) and a somewhat C-shaped (in lateral and mesal views) acropodite (a) longitudinally divided in the distal half into two branches, solenomeral branch (sb) and solenophore (sph). Solenomeral branch positioned mesally, with a narrow “neck”, then abruptly expands and transversely divides into two processes, solenomere (s) and distal solenomeral process (dsp). Extended part of solenomeral branch with spiculiform outgrowths. Solenomere (s) very long, slender, subdistally with a small bifurcation. Distal solenomeral process (dsp) extends in the same direction as solenomere and is half as long as solenomere; bifurcated=with small additional mesal tooth (t). Solenophore (sph) longer and more robust than solenomeral branch, characterized by a robust, lateral, ear-shaped lamella (ll) and a distal projection (dp). Lateral lamella (ll) begins at bifurcation of solenomeral branch and solenophore, surrounding laterally solenophore up to beginning of distal ending; lateral margins of lamella denticulated. Distal projection (dp) with strongly developed, basal lamellar lobe (bl), with mesal thickening (mt) and with relativelly short and acuminate process (ap). Seminal groove (sg) starts from seminal fossa (sf) mesally on prefemorite, extends along mesal side of acropodite up to bifurcation of solenomeral branch and solenophore, then passes on lateral side of solenomeral branch, further on solenomere, ending distally.

###### Habitat.

Saeva dupka Cave is a show cave which is now heavily impacted by electrification and continuous touristic flow. The cave has naturally formed 400 meters of corridors and halls. The samples from the cave were taken in 1997, under stones in clay, when the cave was temporarily closed for visitors due to the change of its governance during the democratic changes in Bulgaria. After more than 20 years of active exploration of the cave, new material needs to be collected to assess whether the species was influenced by the human activities. Saeva dupka Cave is inhabited by numerous and diverse invertebrate taxa, but the only troglobiont currently on record is the local endemic *Bulgariellatranteevi* Z. Karaman, 1958 (Coleoptera, Leiodidae) (Beron 2015).

###### Distribution.

So far known only from two caves in Lovech District (Fig. 18).

###### Remarks.

Based on material from Saeva dupka Cave, Stoev (2004) already noticed that there were certain differences in the structure of gonopods of that sample and *Bacillidesmusdentatus*, and he did not exclude the possibility that it belonged to a new taxon. However, he still treated this as *Bacillidesmusbulgaricusdentatus*. After reviewing the material that was available to him, as well as based on the newly studied material, we describe it above as a new species.

##### ﻿ Balkanodesminus serbicus

Taxon classificationAnimaliaPolydesmidaTrichopolydesmidae

gen. nov. et sp. nov.

8FBCC310-088A-5EAE-90DC-B67E2AF03D7A

http://zoobank.org/2684D82A-21C8-4235-8B2C-39886C7440B1

**Figure 10.**
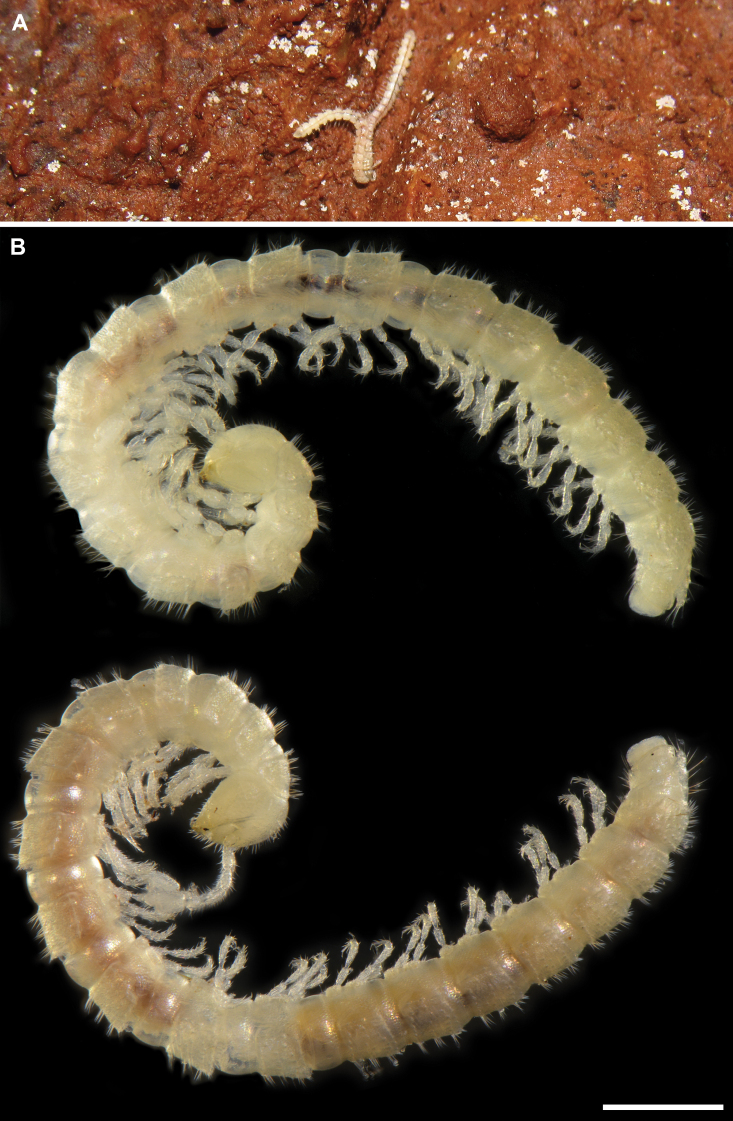
*Balkanodesminusserbicus* gen. nov. et sp. nov., habitus (NHMW) **A** mating in situ (photo D. Antić) **B** holotype ♂ (NHMW MY10262) and paratype ♀ (NHMW MY10265), respectively, lateral views. Scale bar: 0.5 mm.

**Figure 11.**
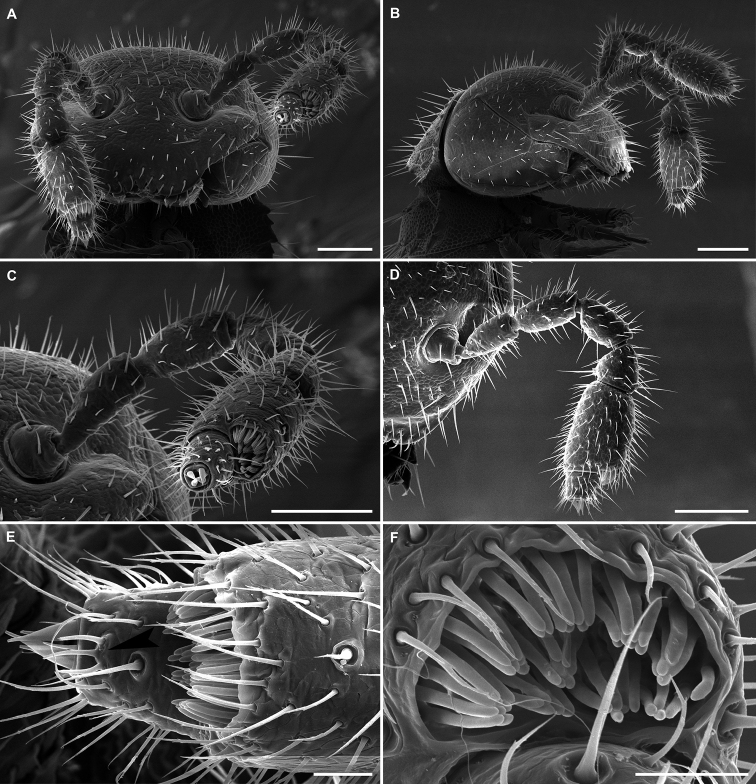
*Balkanodesminusserbicus* gen. nov. et sp. nov., paratype ♀ habitus (NHMW MY 10264) **A** head, anterior view **B** head and collum, lateral view **C, D** left antenna, semi-anterior and anterior views, respectively **E** right antennomeres 6 and 7, dorsal view (arrow indicates small bulge with sensilla basiconica spiniformia) **F** sensilla basiconica of left antennomere 6, disto-dorsal view. Scale bars: 0.1 mm (**A–D**), 0.02 mm (**E, F**).

**Figure 12.**
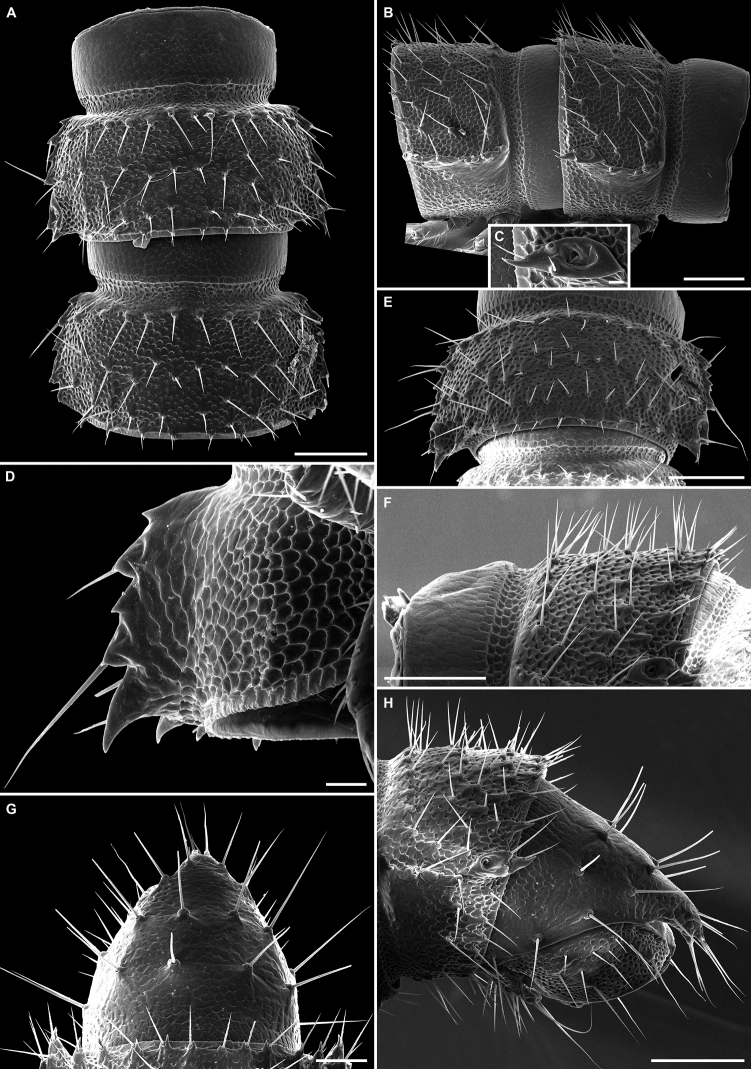
*Balkanodesminusserbicus* gen. nov. et sp. nov., paratype ♀ habitus (NHMW MY10264) **A, B** midbody rings 10 and 11, dorsal and lateral views respectively **C** right ozopore 10, lateral view **D** right half of body ring 4, ventral view **E, F** body ring 17, dorsal and lateral views, respectively **G** telson, dorsal view **H** body ring 18 and telson, lateral view. Scale bars: 0.1 mm (**A, B, E, F, H**), 0.05 mm (**G**), 0.02 mm (**D**), 0.01 mm (**C**).

**Figure 13.**
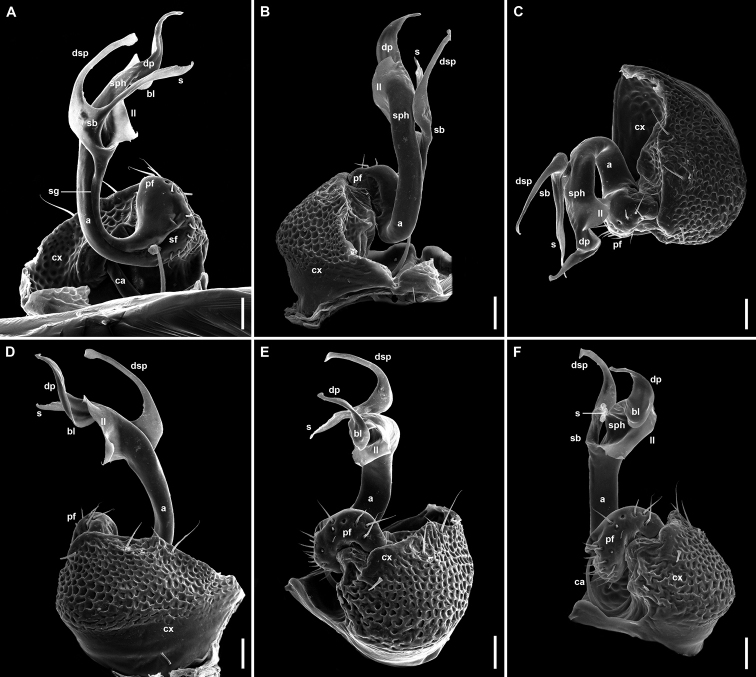
*Balkanodesminusserbicus* gen. nov. et sp. nov., paratype ♂ left gonopod (NHMW MY10263) **A** mesal view **B** anterior view **C** distal view **D** lateral view **E** postro-lateral view **F** posterior view. Abbreviations: **a** acropodite, **bl** basal lamellar lobe of solenophore, **ca** cannula, **cx** coxa, **dp** distal projection of solenophore, **dsp** distal solenomeral process, **ll** lamella of solenophore, **pf** prefemorite, **s** solenomere, **sb** solenomeral branch, **sf** seminal fossa, **sg** seminal groove, **sph** solenophore. Scale bars: 0.02 mm.

Figs 10 , 11 , 12 , 13 , 17J , 18

###### Diagnosis.

Differs from *Balkanodesminusdentatoides* gen. nov. et sp. nov. and *B.dentatus* gen. nov., comb. nov. by the presence of longer (vs. shorter) metatergal setae and their smaller (vs. greater) number of rows, as well as by the presence of more simplified gonopods, with uniramous (vs. biramous) distal solenomeral process and smaller and smooth (vs. larger and denticulated) lamella of solenophore. From *B.bulgaricus* gen. nov., comb. nov., with which it shares similar habitus and similar gonopods, it differs by the presence of smaller (vs. larger) lamella of solenophore, more robust and sigmoid (vs. slender, almost straight) distal projection of solenophore, distal projection with (vs. without) basal lobe, and more robust (vs. slenderer) solenomere, exceeding the distal solenomeral process by ¼ (vs. ½) of its length.

###### Name.

The specific name is an adjective derived from the type locality.

###### Material examined.

***Holotype*** ♂ (NHMW MY10262), Serbia, Niš, Mt. Kalafat, village of Cerje, Cerjanska Cave (= Provalija Cave), 29.X.2017, leg. D. Antić.

***Paratypes*.** 1 ♂ (NHMW MY10263), 1 ♀ (used for SEM, NHMW MY10264), 1 ♀, 7 juveniles (NHMW MY10265), same data as for holotype.

###### Description.

***Number of body rings and measurements***: Body with 19 rings (including telson) in adults, moniliform (Fig. 10). Holotype male and paratype male 4.8 mm and 4.7 mm long, respectively; width of midbody pro- and metazonae 0.30 mm and 0.45 mm, respectively. Paratype females 5.0 mm and 5.2 mm long, width of midbody pro- and metazonae 0.35 mm and 0.50 mm, respectively.

***Coloration***: Entirely pallid, slightly translucent (Fig. 10).

***Head***: Broader than collum, setose; epicranial suture poorly developed; isthmus between antennae ≈ 1.7 × broader than diameter of antennal socket (Fig. 11A, B). Labrum with three labral teeth, and with 3+3 labral and five supralabral setae (Fig. 11A). Gnathochilarium without peculiarities. Antennae rather short, clavate (Fig. 11). Antennomere length 6 > 2 = 3 = 4 > 5 > 7 = 1. Antennae 0.7 mm long in the holotype male; length/breadth ratios of antennomeres 1–7: 1 (1), 2 (2), 3 (2), 4 (2), 5 (1), 6 (1.5) and 7 (1). Antennomere 6 with four sensilla trichoidea and with strongly developed disto-dorsal pit with numerous long sensilla basiconica partially exposed outside the pit (Fig. 11E, F). Antennomere 7 with one sensillum trichodeum and a small bulge with three sensilla basiconica spiniformia (Fig. 11E). Four apical cones (Fig. 11C).

***Collum***: Semi-circular, with one or two lateral incisions and ≈ 5 irregular rows of relatively long and trichoid setae.

***Body rings***: Tegument shining, texture alveolate, reticulate and scaly. Setae relatively long and trichoid, originating from small tubercules (Figs 10B, 12). Posteriormost tubercules mostly with a small thorn (Fig. 12A, E). Rings 2–4 with three mostly regular rows of setae, one anterior and two posterior (Fig. 10B). Rings 5–18 with ≈ 4–6 irregular rows of setae (Figs 10B, 12). Paraterga serrated, with four or five teeth (Fig. 12A, D, E). Pore formula normal: 5, 7, 9, 10, 12, 13, 15–18. Poriferous metazonae with an enlarged posterolateral cone bearing an ozopore and three medium-sized setae (Fig. 12C). Epiproct blunt, directed slightly ventrad (Fig. 12G, H). Paraprocts semi-spherical, each with 2 long setae originating from small tubercules and ≈ 10 shorter setae without tubercules (Fig. 12H). Hypoproct trapeziform, with two long distal setae and numerous shorter setae throughout (Fig. 12H). Sterna unmodified, poorly setose. Pleurosternal carinae absent, only a few small teeth sometimes present on rings 2 and 3. Gonopod’s aperture large, subsemi-circular.

***Walking legs***: Legs 1–3 in males with swollen femur; coxa 2 with a short mesal apophysis (cf. Strasser 1966a: 341, fig. 13). No other peculiarities.

***Gonopods*** (Figs 13, 17J): Coxa (cx) large, semi-circular in ventral and lateral views, with differentiated gonocoel mesally; lateral part swollen, alveolate, with three long setae near mesal ridge. Cannula (ca) long, C-shaped. Telopodite long compared to coxa, consisting of a transverse, setose prefemorite (pf) and a somewhat C-shaped (in lateral and mesal views) acropodite (a) longitudinally divided in its distal half into two branches, solenomeral branch (sb) and solenophore (sph). Solenomeral branch positioned mesally, with a narrow base, then abruptly expands and transversely divides into two processes, solenomere (s) and distal solenomeral process (dsp). Solenomere (s) long, slender, distally expanded (in lateral and mesal views), forming U-shaped rift with distal solenomeral process. Distal solenomeral process (dsp) extends in the same direction as solenomere, ¾ the length of solenomere; ending with a small expansion (in lateral and mesal views). Solenophore (sph) longer and more robust than solenomeral branch, characterized by a lateral, ear-shaped lamella (ll) and a distal projection (dp). Lateral lamella (ll) begins at bifurcation of solenomeral branch and solenophore, surrounding laterally solenophore up to beginning of distal projection; lateral margins of lamella smooth. Distal projection (dp) sigmoid (in lateral and mesal views), with well-developed, basal lamellar lobe (bl). Seminal groove (sg) starts from seminal fossa (sf) mesally on prefemorite, extends along mesal side of acropodite up to bifurcation of solenomeral branch and solenophore, then passes on lateral side of solenomeral branch, further on solenomere, ending subdistally.

###### Habitat.

With its 6131 m of explored channels, the Cerjanska Cave represents one of the longest and most significant fluviokarst underground systems in Serbia. This is a relatively simple speleological object, consisting of one main river channel in two levels with a length of 4903 m, as well as several side channels with a total length of 1228 m (Nešić 2016). Numerous arthropod taxa have been registered in the cave, from epigean, guanophiles, trogloxenes, and troglophiles to troglobionts (Pavićević et al. 2016). The troglobionts include the endemic Balkan harvestman *Paranemastomabureschi* (Roewer, 1926), the millipede *Dazbogosomanaissi* Makarov & Ćurčić in Makarov et al. 2012, and the carabid beetle *Duvaliusrtanjensisprovalijae* Pavićević, Zatezalo & Popović, 2016, the latter two endemics of Cerjanska Cave.

Despite many years of speleological and biospeleological research in the Cerjanska Cave, the new taxon was not registered until the first Biospeleological Expedition of the Serbian Biospeleological Society, organized at the end of October 2017. All 11 specimens were found in a small area, in the initial part of the cave. One male, one female and seven juveniles were found on the left side of the river, on a small branch of a tree lying on the wet sand. Another male and female were found just on the opposite side of the river, on the wall, in copulation (Fig. 10A).

###### Distribution.

So far, known only from its type locality, the Cerjanska Cave, Serbia (Fig. 18).

###### Remarks.

This is the first representative of the family Trichopolydesmidae in Serbia.

#### Taxa from the Rhodope Mountains

##### ﻿Genus Rhodopodesmus gen. nov.

Taxon classificationAnimaliaPolydesmidaTrichopolydesmidae

7E912988-7CD0-5E1A-8926-D16F67DCF5F2

http://zoobank.org/1D609274-0185-4A6E-85CF-D32654CBFEF9

###### Type species.

*Rhodopodesmusniveus* gen. nov. et sp. nov., by monotypy.

###### Diagnosis.

Differs from all European Trichopolydesmidae by the presence of characteristic acropodite of the gonopods divided into two branches that are parallel and completely meso-laterally oriented to each other, with solenomeral branch transversely tripartite, where the proximal-most branch is the shortest, while solenomere and distal solenomeral process are longer and of the same length. The most similar genus is *Balkanodesminus* gen. nov., but it differs from *Rhodopodesmus* gen. nov., by the presence of bifid solenomeral branch (for more details on gonopod differences see below under Remarks).

In addition, the diagnosis can be amended with the following combination of characters: small size (4.3–5.4 mm), 19 body rings (including telson), sensilla basiconica on antennomere 6 partially exposed outside the pit, hypoproct with more than two long distal setae, paraprocts with more than 2+2 long setae, metaterga with 4–8 irregular rows of medium-sized trichoid setae.

###### Name.

The new genus is named after the Rhodope Mountains, its type locality, in combination with –*desmus*, the common suffix in Polydesmida. The name is a masculine noun.

##### ﻿ Rhodopodesmus niveus

Taxon classificationAnimaliaPolydesmidaTrichopolydesmidae

gen. nov. et sp. nov.

B925ACBE-B25B-532F-A3DC-5C4A5F3C16EA

http://zoobank.org/CC18A689-89BD-4197-922D-9CB3D8B3EA77

**Figure 14.**
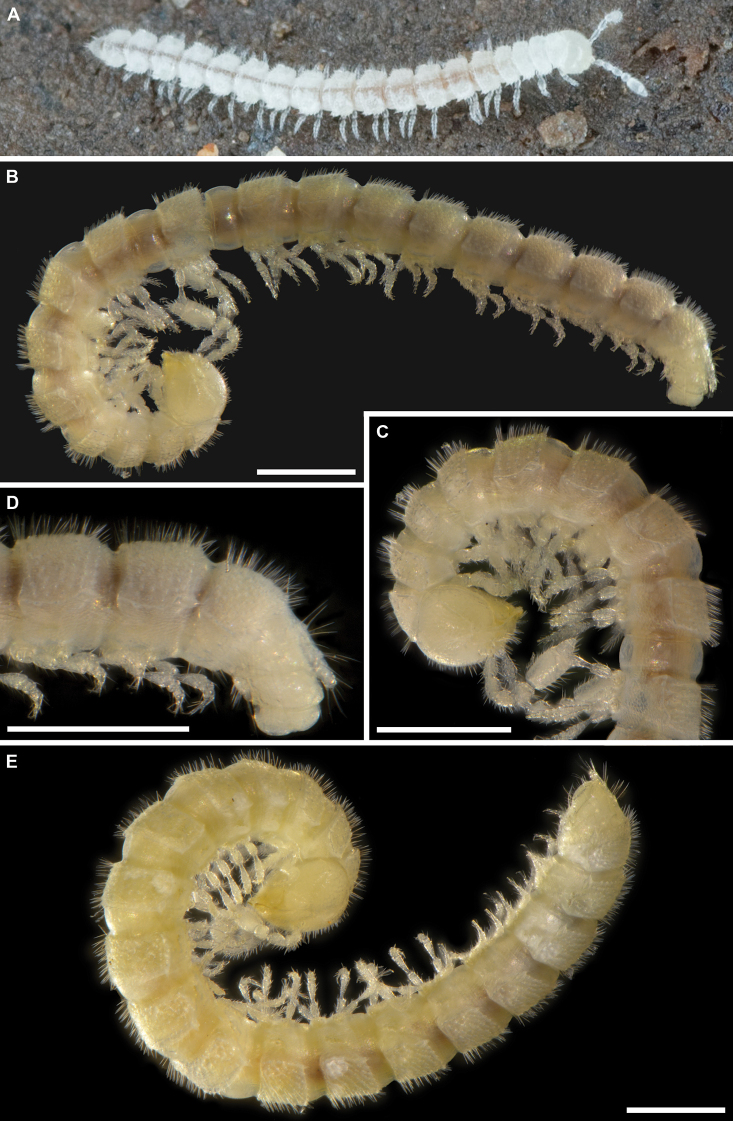
*Rhodopodesmusniveus* gen. nov. et sp. nov., holotype ♂ (**A–D**, NMNHS-10823) and paratype ♀ (**E**, NHMW MY10259) habitus **A** in situ, dorsal view (photo H. Reip) **B** lateral view **C** anterior part of body, lateral view **D** posterior part of body, lateral view **E** lateral view. Scale bars: 0.5 mm.

**Figure 15.**
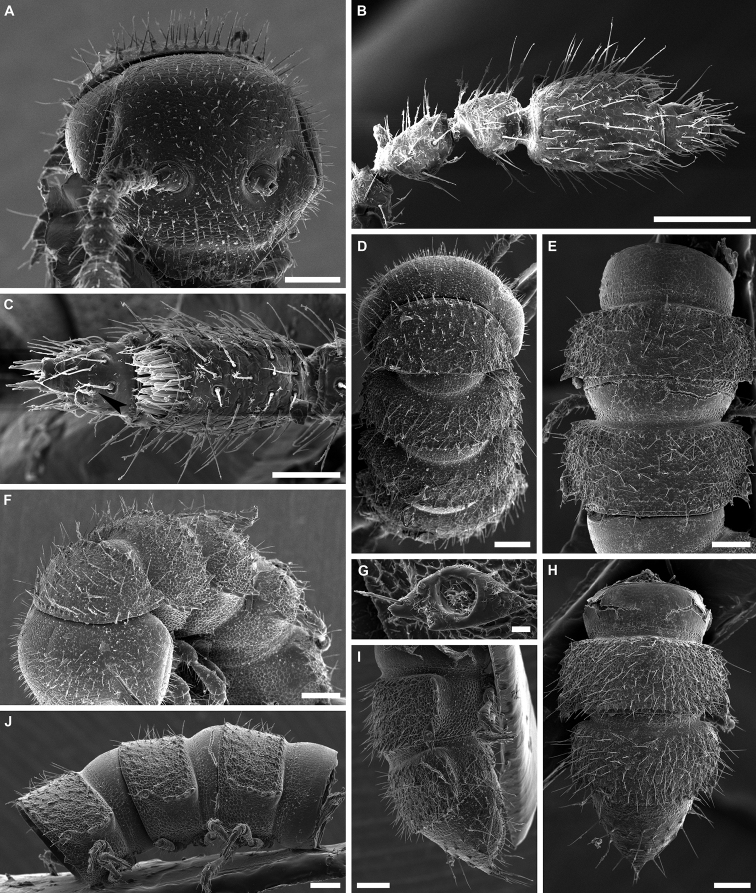
*Rhodopodesmusniveus* gen. nov. et sp. nov., paratype ♀ habitus (NHMW MY10259) **A** head, anterior view **B** right antenna, lateral view **C** tip of right antenna, dorsal view (arrow indicates small bulge with sensilla basiconica spiniformia) **D** anterior part of body, dorsal view **E** body rings 8 and 9, dorsal view **F** anterior part of body, dorso-lateral view **G** right ozopore 9, lateral view **H, I** body rings 17–19, dorsal and lateral views, respectively **J** body rings 8–10. Scale bars: 0.1 mm (**A, B, D–F, H–J**), 0.05 mm (**C**), 0.02 mm (**G**).

**Figure 16.**
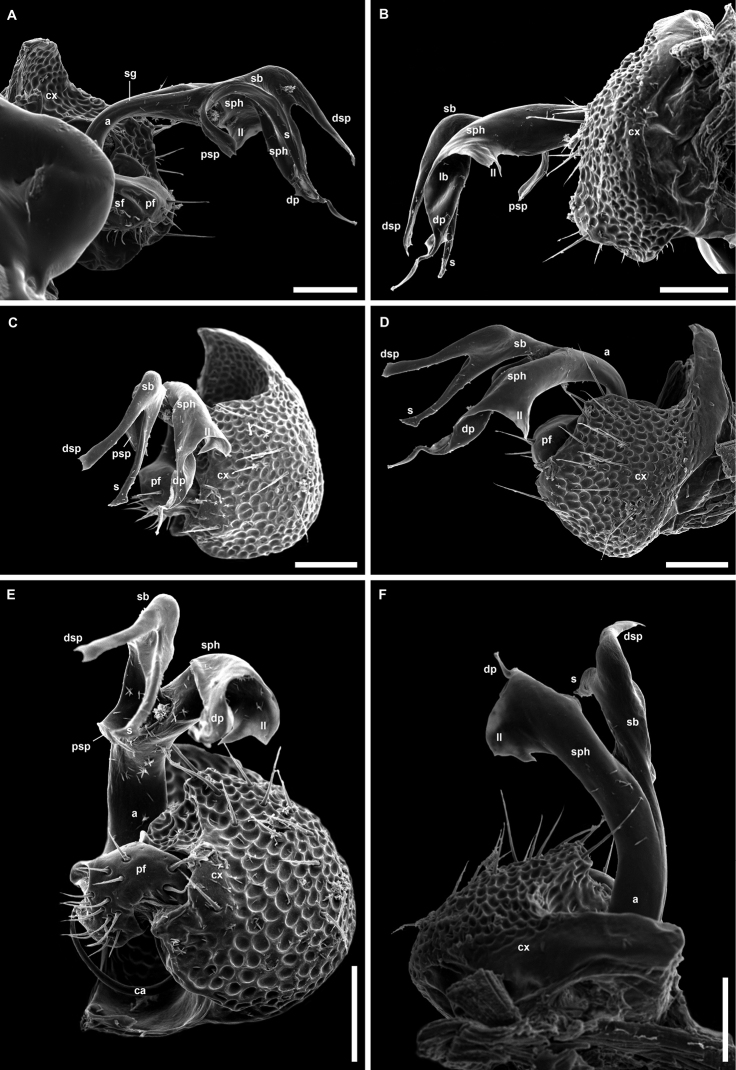
*Rhodopodesmusniveus* gen. nov. et sp. nov., holotype ♂ left gonopod (NMNHS-10823) **A** mesal view **B** lateral view **C** disto-lateral view **D** antero-lateral view **E** postero-distal view **F** anterior view. Abbreviations: **a** acropodite, **ca** cannula, **cx** coxa, **dp** distal projection of solenophore, **dsp** distal solenomeral process, **ll** lamella of solenophore, **pf** prefemorite, **psp** proximal solenomeral process, **s** solenomere, **sb** solenomeral branch, **sf** seminal fossa, **sg** seminal groove, **sph** solenophore. Scale bars: 0.05 mm.

**Figure 17.**
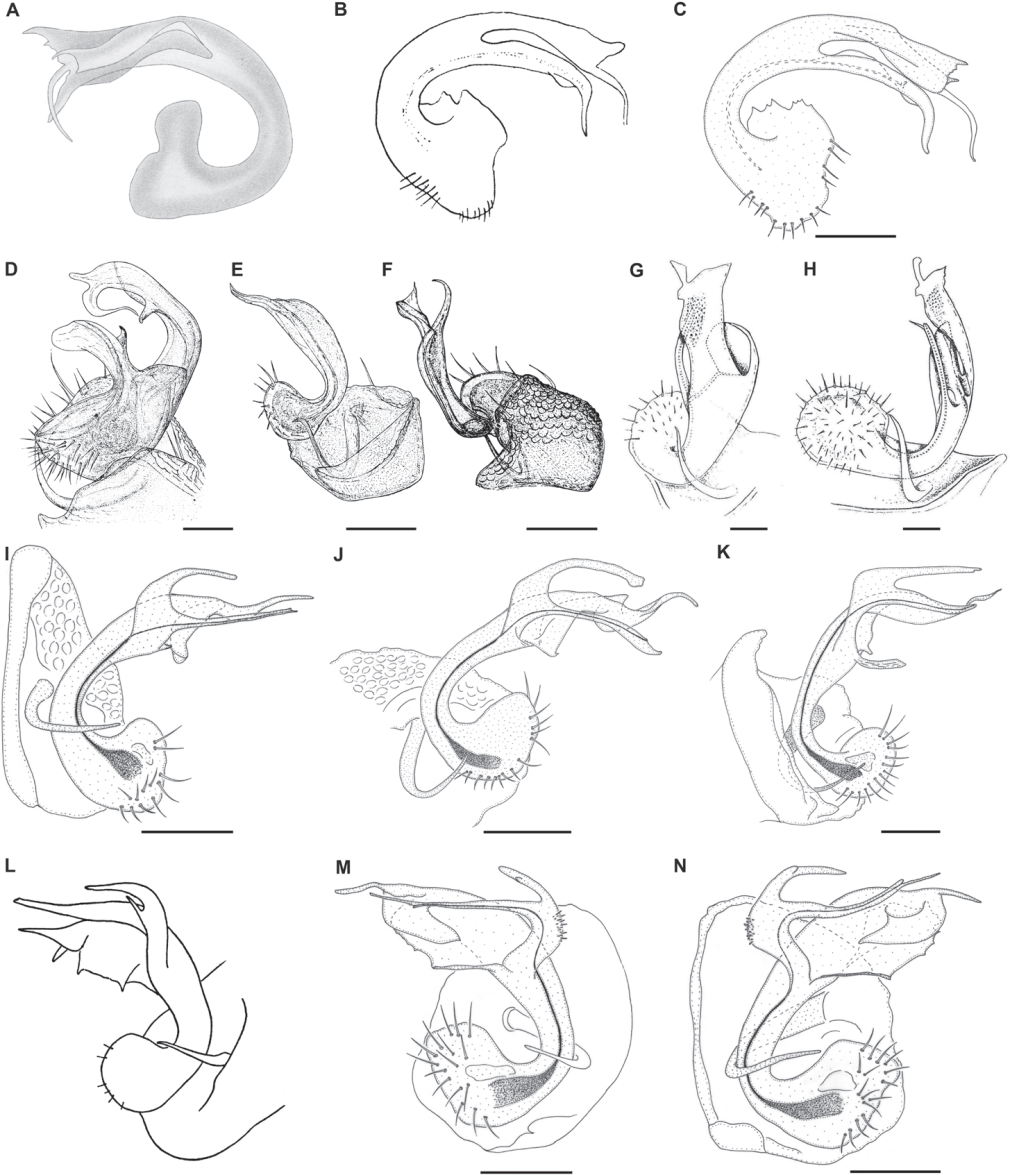
Gonopods of cavernicolous Trichopolydesmidae from the Carpathian-Balkan arch and Rhodopes **A–C***Bacillidesmusfiliformis* (Latzel, 1884) lectotype ♂ (NHWM MY3754) **A** after Attems (1898)**B** ?left gonopod, ?mesal view, after Attems (1940)**C** ?left gonopod, ?mesal view, present study **D***Banatodesmusjeanneli* (Tabacaru, 1980) syntype ♂, right gonopod, mesal view, after Tabacaru (1980)**E, F***Napocodesmusflorentzae* Tabacaru, 1975 holotype ♂, right gonopod, mesal and anterior views, respectively, after Tabacaru (1975)**G, H***Trichopolydesmuseremitis* Verhoeff, 1898 ♂ from Cloşani cave, right gonopod, antero-mesal and mesal views, respectively, after Ceuca (1958)**I***Balkanodesminusbulgaricus* (Strasser, 1962) gen. nov., comb. nov. ♂ from Ponora Cave (NMNHS-10813), left gonopod, mesal view **J***Balkanodesminusserbicus* gen. nov. et sp. nov. paratype ♂ (NHMW MY10263), left gonopod, mesal view **K***Rhodopodesmusniveus* gen. nov. et sp. nov. holotype ♂ (NMNHS-10823), left gonopod, mesal view **L***Balkanodesminusdentatus* (Strasser, 1966a) gen. nov., comb. nov., stat. nov. syntype ♂, right gonopod, mesal view, after Strasser (1966a)**M***Balkanodesminusdentatoides* gen. nov. et sp. nov. paratype ♂ (NHMW MY10258), right gonopod, mesal view **N***Balkanodesminusdentatoides* gen. nov. et sp. nov. ♂ from Sopotska Cave (NHMW MY10267), left gonopod, mesal view. Scale bars: 0.05 mm.

**Figure 18.**
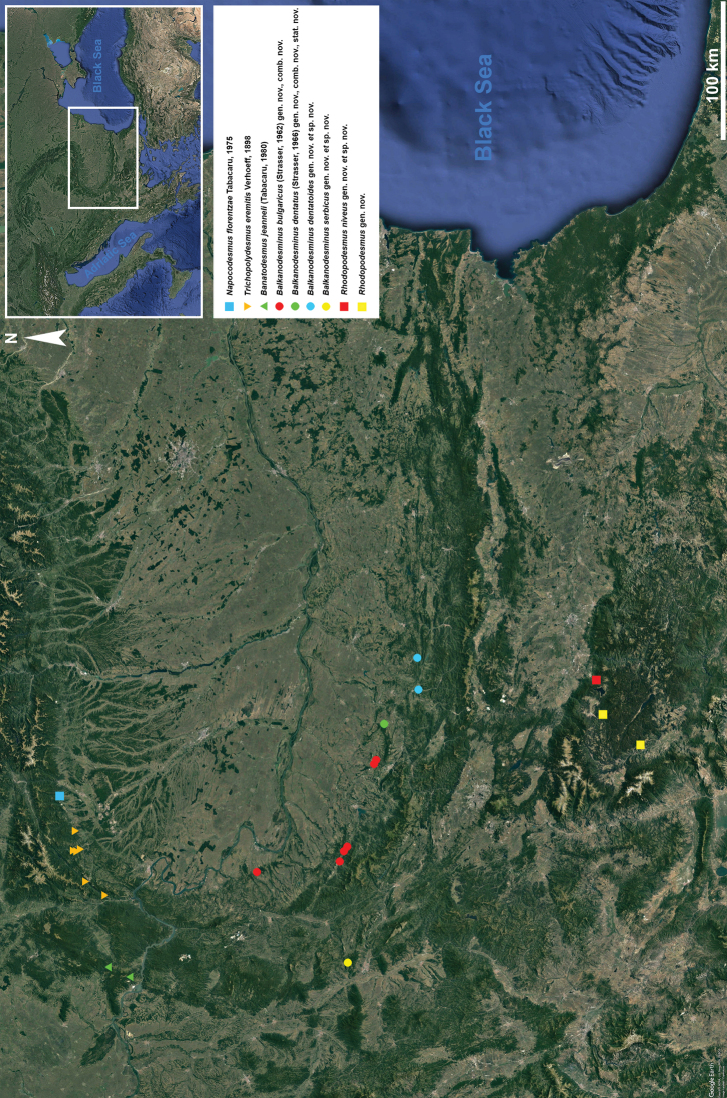
Distribution of cavernicolous Trichopolydesmidae from the Carpathian-Balkan arch and the Rhodope Mountains.

Figs 14 , 15 , 16 , 17K , 18

Bacillidesmus sp. nov.—Vagalinski and Stoev 2011: 135.
Bacillidesmus sp. [nov.]—Beron 2015: 80.

###### Diagnosis.

As for the monospecific genus.

###### Name.

The specific name is a Latin adjective; *niveus* refers to the snow-white body color of the living specimens. Furthermore, the name of the type locality, cave Snezhanka, in Bulgarian means Snow White, the heroine from the fairy tale of the Brothers Grimm.

###### Material examined.

***Holotype*** ♂ (NMNHS-10823), Bulgaria, Pazardzhik District, Peshtera Municipality, Peshtera, Snezhanka Cave, N 42.00222, E 24.27764, 26.X.2020, leg. D. Antić & B. Vagalinski.

***Paratypes*.** 3 ♀♀ (one used for SEM, NMNHS-10824–10826), same data as for holotype; 1 ♀ (used for SEM, NHMW MY10259) same data as for holotype.

###### Additional material.

1 ♂ (fragments, one gonopod available, NMNHS-10827), 1 ♀ (fragments, NMNHS-10828), both fragments, 1 whole juvenile (NMNHS-10829), same cave but 18.IX.2005, leg. P. Beron.

###### Description.

***Number of body rings and measurements***: Body with 19 rings (including telson) in adults, moniliform (Fig. 14). Holotype male 5.3 mm long, width of midbody pro- and metazonae 0.35 mm and 0.50 mm, respectively. Paratype females 4.3–5.4 mm long, width of midbody pro- and metazonae 0.35–0.40 mm and 0.45–0.60 mm, respectively.

***Coloration***: Living animals snow white (Fig. 14A), slightly translucent. Yellowish in alcohol (Fig. 14B–E).

***Head***: Broader than collum, setose; epicranial suture poorly developed; isthmus between antennae ≈ 1.7 × broader than diameter of antennal socket (Fig. 15A, D). Labrum with three labral teeth, and with 3+3 labral and five supralabral setae (Fig. 15A). Gnathochilarium without peculiarities. Antennae rather short, clavate (Figs 14A, C, 15B, C). Antennomere length 6 > 2 = 3 = 4 > 5 > 7 = 1. Antennae 0.8 mm long in holotype male; length/breadth ratios of antennomeres 1–7: 1 (1), 2 (2), 3 (2), 4 (2), 5 (1), 6 (1.5) and 7 (1). Antennomere 6 with four sensilla trichodea and with strongly developed disto-dorsal pit with numerous long sensilla basiconica partially exposed outside the pit (Fig. 15C). Antennomere 7 with one sensillum trichoideum and a small bulge with three sensilla basiconica spiniformia (Fig. 15B, C). Four apical cones (Fig. 15C).

***Collum***: Semi-circular, with one or two lateral incisions and ≈ 6 irregular rows of medium sized and trichoid setae (Fig. 15D, F).

***Body rings***: Tegument shining, texture alveolate, reticulate and scaly. Rings densely setose (Fig. 14B–E). Setae medium sized and trichoid, originating from small tubercules (Fig. 15D–F, H–J). Posteriormost tubercules mostly with a small thorn (Fig. 15E). Rings 2–4 with ≈ 4 rows of setae (Fig. 15D, F). Rings 5–18 with ≈ 6–8 irregular rows of setae (Fig. 15D–F, H–J). Paraterga serrated, with 5–7 teeth (Fig. 15E, H). Pore formula normal: 5, 7, 9, 10, 12, 13, 15–18. Poriferous metazonae with enlarged posterolateral cone bearing an ozopore and three medium-sized setae (Fig. 15G). Epiproct triangular in dorsal view, directed slightly caudoventrad (Fig. 15H, I). Paraprocts semi-spherical, each with two long setae originating from small tubercules and ≈ 10 shorter setae without tubercules (Fig. 15I). Hypoproct trapeziform, with two long distal setae and numerous shorter setae throughout (Fig. 15I). Sterna unmodified, poorly setose. Pleurosternal carinae absent, only a few small teeth present on rings 2 and 3 (Fig. 15F). Gonopod aperture large, subsemi-circular.

***Walking legs***: Legs 1–3 in males with swollen femur, especially femur 2; coxa 2 with a short mesal apophysis (cf. Strasser 1966a: 341, fig. 13). No other peculiarities.

***Gonopods*** (Figs 16, 17K): Coxa (cx) large, semi-circular in ventral view, with deep gonocoel mesally; anterior third much lower than rest of coxa, shield-like, thus lateral, swollen and alveolate part rectangular in lateral view; with ≈ 15 setae. Cannula (ca) long, C-shaped. Telopodite long compared to coxa, consisting of a transverse, setose prefemorite (pf) and more or less C-shaped (in lateral and mesal views) acropodite (a) longitudinally divided in distal half into two branches, solenomeral branch (sb) and solenophore (sph). Solenomeral branch positioned mesally, transversely divided into three processes, besides solenomere (s) and distal solenomeral process (dsp), there is an additional, proximal solenomeral process (psp), more or less spatulate and forming C-shaped rift with solenomere. Both solenomere (s) and distal solenomeral process (dsp) long, slender, of same length, forming acute angle at bifurcation. Solenophore (sph) longer than solenomeral branch, characterized by a lateral lamella (ll) and a distal projection (dp). Lateral lamella (ll) with triangular lobe. Distal projection (dp) long, thin and twisted. Seminal groove (sg) starts from seminal fossa (sf) mesally on prefemorite, extends along mesal side of acropodite up to bifurcation of solenomeral branch and solenophore, then passes on lateral side of solenomeral branch, further proximally on solenomere, ending distally.

###### Habitat.

Snezhanka Cave consists of a single gallery forming six distinct halls with total length of 348 m. The entrance is located at 865 m a.s.l. The cave is rich in diverse sinter formations and sinter ponds. It was established as a natural monument in 1961, and has served as show cave since 1968 (Petrov and Stoev 2007). Most of the cave’s invertebrate fauna known at present includes either trogloxenes or troglophiles (Beron 2015), with the exception of the local endemic *Paralovriciaberoni* Giachino, Guéorguiev & Vailati, 2011 (Coleoptera, Carabidae), which is considered a probable hypogean, although not typical troglobitic species (Giachino et al. 2011). Another myriapod known from this cave is *Lithobiuslakatnicensis* Verhoeff, 1926.

All five recently collected specimens of *Rhodopodesmusniveus* gen. nov. et sp. nov. by D.A. and B.V. were found in the middle part of the cave at two spots, and all were in rotten wood.

###### Distribution.

So far, known only from its type locality, the Snezhanka Cave, Bulgaria (see also under Remarks) (Fig. 18).

###### Remarks.

As mentioned above, it seems that the most similar genus to *Rhodopodsemus* gen. nov. is *Balkanodesminus* gen. nov., which makes sense due to their distributions. These two genera share not only similarities in certain habitus features but also in the gonopods. Both include small-bodied species with 19 rings in adults, with sensilla basiconica on antennomere 6 partially exposed outside the pit, hypoproct with more than two long distal setae and paraprocts with more than 2+2 long setae. The conformation of the gonopods is very similar, where the acropodite is longitudinally divided into two branches, with the solenomeral branch transversely divided into long and relatively slender solenomere and well-developed distal process in both genera. Based on this, both genera differ from other European Trichopolydesmidae. However, in *Rhodopodesmus* gen. nov. the solenomeral branch is trifid, where in addition to solenomere and distal process, in the base of this branch there is another, proximal process, which is more or less spatulate. These two genera also differ in some details of the gonopod coxa. *Rhodopodesmus* gen. nov. has a very deep gonocoel, i.e., the anterior third of the coxa is much lower than the rest it, in the form of a shield, so that the rest of the coxa has a more or less rectangular shape laterally, while in *Balkanodesminus* gen. nov. it is semi-circular. Also, the coxa in *Rhodopodesmus* gen. nov. has circa 15 setae, while the same in species of *Balkanodesminus* gen. nov. bears only 3.

It is worth mentioning that specimens of *Rhodopodesmus* gen. nov. were found in two more caves in the Rhodopes, viz., one female in Dupkata (= Dupcheto) Cave near Rakitovo and one male and one female in Skoka Cave near Ribnovo. Unfortunately, the material from Skoka Cave (including one male) could not be relocated in NMNHS for this study, and we still do not know whether it is *R.niveus* gen. nov. et sp. nov. or a new species. The female from Dupkata Cave probably belongs to *R.niveus* gen. nov. et sp. nov. considering its proximity to the type locality of this species. Both localities are mapped (Fig. 18, yellow squares), and already noted under *Bacillidesmus* sp. nov. by Vagalinski and Stoev (2011).

### ﻿Key to the cavernicolous genera of Trichopolydesmidae from the Carpathian-Balkan arch and the Rhodope Mountains (including *Bacillidesmus*)

**Table:** 

1	Adults with 20 body rings (including telson)	**2**
–	Adults with 19 body rings (including telson)	**3**
2	Acropodite of the gonopods trifid, with slender solenomere	***Trichopolydesmus* (Fig. 17G, H)**
–	Acropodite of the gonopods bifid, with enlarged, oval, paddle-like solenomere	***Banatodesmus* (Fig. 17D)**
3	Sensilla basiconica completely enclosed inside the pit of antennomere 6	***Bacillidesmus* (Fig. 17A–C)**
–	Sensilla basiconica partially exposed outside the pit of antennomere 6	**4**
4	Posterior cones of metaterga hook-shaped. Solenomeral branch of acropodite simple, only with solenomere	***Napocodesmus* (Fig. 17E, F)**
–	Posterior cones of metaterga not hook-shaped. Solenomeral branch of acropodite transversely bifid or trifid	**5**
5	Gonopod coxa with ≈ 15 setae. Solenomeral branch of acropodite transversely trifid	***Rhodopodesmus* gen. nov. (Fig. 17K)**
–	Gonopodal coxa with three setae. Solenomeral branch of acropodite transversely bifid	***Balkanodesminus* gen. nov. (Fig. 17I, J, L–N)**

To distinguish easily all six genera and nine species see Fig. 17.

#### Additional material examined

##### ﻿ Cottodesmus crissolensis

Taxon classificationAnimaliaPolydesmidaTrichopolydesmidae

Verhoeff, 1936

60EF76CA-42B1-58B6-8B29-7CA21748E51A

###### Material examined.

***Syntype*** ♀ (NHMW MY3760), Italy, Kottische Alpen [Cottian Alps], Monte Viso, oberhalb [above] Crissolo, 1300–1500 m a.s.l., 2–3.10.1932, leg. K. Verhoeff, don. Verhoeff 01.VII.1940.

##### ﻿ Haplocookia enghoffi

Taxon classificationAnimaliaPolydesmidaTrichopolydesmidae

Akkari & Mauriès, 2018

BEA2A007-0852-5DD2-8878-9795C0E05EFA

###### Material examined.

***Paratype*** ♂ (NHMW MY9367), Tunisia, Cap Bon peninsula, Nabeul district, Jebel Abderrahman, 28.XI.2004, leg. N. Akkari.

##### ﻿ Heterocookia tunisiaca

Taxon classificationAnimaliaPolydesmidaTrichopolydesmidae

Ceuca, 1967

53D52FD4-A1C5-5323-A142-23592AD712C6

###### Material examined.

1 ♂ (NHMW MY9992), Tunisia, Ariana Governorate, El Ghazela, garden, under stones, 24.XI.2003, leg. N. Akkari.

## ﻿Discussion

The family Trichopolydesmidae, as accepted today (see Golovatch et al. 2018, 2022), includes ≈ 100 genera and > 220 species, which are mostly distributed in the Northern Hemisphere. Unfortunately, the family cannot be clearly defined and diagnosed, as it includes a wide range of taxa that differ significantly not only in appearance, but also in gonopod conformation. However, according to Tabacaru and Giurginca (2016), this family is composed of exclusively European genera: *Trichopolydesmus* (monospecific), *Bacillidesmus* (monospecific), *Cottodesmus* Verhoeff, 1936 (2 species), *Galliocookia* Ribaut, 1955 (4 species), *Verhoeffodesmus* Strasser, 1959 (monospecific), *Occitanocookia* Mauriès, 1980 (monospecific), *Caucasodesmus* (5 species), *Napocodesmus* (2 species), *Banatodesmus* (monospecific), *Balkanodesmus* Antić & Reip in Antić et al. 2014 (monospecific), *Velebitodesmus* Antić & Reip in Antić et al. 2014 (monospecific), and *Solentanodesmus* Antić & Reip in Antić et al. 2014 (monospecific). The largest number of these genera, eight of them, viz., *Trichopolydesmus*, *Bacillidesmus*, *Verhoeffodesmus*, *Napocodesmus*, *Banatodesmus*, *Balkanodesmus*, *Velebitodesmus*, and *Solentanodesmus* are from the Balkans, and *Caucasodesmus* from the Caucasus and Crimea. With the addition of two new genera, *Balkanodesminus* gen. nov. and *Rhodopodesmus* gen. nov., they all seem to form a natural group characterized by the following combination of characters: metatergal setae always trichoid, in most cases on small tubercles arranged almost always in more than four irregular transverse rows, antennomere 5 without distodorsal sensilla basiconica, antennomere 6 always with sensilla basiconica in a distodorsal sensory pit, completely or partially concealed inside, gonopod telopodite relatively long compared to gonopod coxa, basal part of prefemorite transverse to the body axis, acropodite uni-, bi- or triramous, always without seminal vesicle or pulvillus. Chiefly for the purpose of this discussion we will call this group “true” trichopolydesmids. The other three genera considered in Trichopolydesmidae sensu Tabacaru and Giurginca (2016), viz., *Cottodesmus*, *Galliocookia*, and *Occitanocookia*, also show some affinities with this group, but differ in a number of important characters. Both species of the genus *Cottodesmus* are characterized by bacilliform rather than trichoid setae on metaterga. Although Verhoeff (1936) described *C.crissolensis* Verhoeff, 1936 with trichoid setae, after examination of the female syntype, it becomes clear that the setae are bacilliform and arranged in four almost regular rows. Further, at least *C.crissolensis* is characterized by sensilla basiconica on both antennomeres 5 and 6, with no sensory pits in both cases. Similar to *Cottodesmus*, the genus *Occitanocookia* is characterized by sensilla basiconica on antennomeres 5 and 6, but with trichoid metatergal setae arranged in several irregular rows, as in the “true” trichopolydesmids. Furthermore, the latter genus shows gonopodal prefemorites that are set transverse to the body axis, and a somewhat curved acropodite, both characters observed in the “true” trichopolydesmids. The species *Galliocookiagracilis* Golovatch, 2011, found on Rhodes (very far from the remaining distribution area of the genus in France), possesses sensilla basiconica on antennomeres 5 and 6, while its other three congeners lack sensilla on antennomere 5, like in “true” trichopolydesmids. The generic assignment of the species described from Rhodes, Greece to *Galliocookia* is in our view questionable. In any case, the relatively small gonopodal coxa and prefemorite – which is almost coaxial with the acropodite – places the genus quite distant from the above-mentioned group.

In addition to these 14 genera of (autochthonous?) European Trichopolydesmidae, the recently established genus *Simplogonopus* Vagalinski, Golovatch, Akkari & Stoev, 2019, known from the Balkan mainland and Aegean islands, is classified in Trichopolydesmidae sensu Golovatch (2013) and Golovatch et al. (2018) (Vagalinski et al. 2019). This genus clearly belongs to Afrotropical trichopolydesmids, which are significantly different from the “true” trichopolydesmids from Europe in the presence of exclusively bacilliform metatergal setae distributed mainly in three regular transverse rows, distodorsal sensilla basiconica on antennomeres 5 and 6, but antennomere 6 without distodorsal pit and with relatively small gonopod telopodites, almost completely concealed by very large coxae. The genus *Simplogonopus* and all Afrotropical trichopolydesmids appear to form one natural group (cf. Golovatch et al. 2019, 2022) whose representatives were once assigned to the family Fuhrmannodesmidae.

Two more Mediterranean genera from North Africa are often attributed to the family Trichopolydesmidae as well, viz., *Heterocookia* Silvestri, 1898 and *Haplocookia* Brölemann, 1915 (Akkari and Mauriès 2018). However, these two genera differ from the “true” trichopolydesmids in the presence of three regular transverse rows of bacilliform metatergal setae, and in having both antennomeres 5 and 6 with a distodorsal group of sensilla basiconica, and in showing both gonocoxa and prefemorite rather small. These two latter taxa, as well as the above mentioned genus *Galliocookia*, have been attributed to the family Polydesmidae by Hoffman (1980). It is obvious that the current classification of Trichopolydesmidae is very chaotic and cannot be a ground for any phylogenetic approaches. Some authors believe that the family should be reduced to the European genera only, others assign to it also the North African genera or consider it in a broader sense, including many taxa once classified in the family Fuhrmannodesmidae (Golovatch 2013; Golovatch et al. 2018, 2022). The composition and the relationships of the taxa of Trichopolydesmidae could be resolved only after applying combined morphological and molecular phylogenetic methods. Until then, and relying on morphological characters hitherto applied to the classification of the group only, we tend to believe that Trichopolydesmidae should be restricted to the European genera sensu Tabacaru and Giurginca (2016), with the addition of the genera *Balkanodesminus* gen. nov. and *Rhodopodesmus* gen. nov. described above. The genera of what was previously considered as the family Fuhrmannodesmidae seem to form several natural groups (cf. Hoffman 1980; Golovatch 2011), while some other genera (*Heterocookia* and *Haplocookia*) might well be placed in Polydesmidae. Opinions on the classification of Trichopolydesmidae are highly subjective and, as emphasized earlier, only an integrative approach using molecular markers might clarify the picture in the future.

The Balkan Peninsula, including the southern Carpathians, is obviously a hotspot for the family Trichopolydesmidae, with as many as ten “true” trichopolydesmid genera known mostly from caves in the Balkans. All ten genera are characterized by four or more irregular rows of trichoid setae (except for *Bacillidesmus* with four regular rows), absence of sensilla basiconica on antennomere 5, presence of a distodorsal pit with sensilla basiconica on antennomere 6, as well as gonopods with a relatively transverse basal part of the prefemorite. The genus *Caucasodesmus*, known from caves in the north Caucasus and the Crimean Peninsula (see Golovatch 1985, 2011; Golovatch and VandenSpiegel 2015, 2017; Turbanov et al. 2018), also agrees with this combination of characters. The only exception is *C.inexpectatus* Golovatch, 1985, which has three rows of trichoid setae.

Two morphological clusters of Trichopolydesmidae could be recognized among the taxa inhabiting the Balkan Peninsula: the Dinaric and the Carpathian-Balkan-Rhodopean ones. All taxa described from the Dinarides are characterized by well-developed and denticulate pleurosternal carinae on rings 2–18 (see Antić et al. 2014), while taxa from the Carpathian-Balkanids and Rhodopes have only a few pleurosternal teeth on anterior rings (mainly on rings 2–4). Eight of ten Balkan genera are monospecific, including the genus *Verhoeffodesmus*, although Strasser (1959, 1966b) described two species (see Antić et al. 2014). Again, the Balkan Peninsula proves to be a prominent hotspot of millipede diversity in Europe. This primarily concerns the cave fauna, with trichopolydesmids being no exception. Better equipment and more manpower in the recent years have contributed to the discovery of interesting taxa, also from this group, primarily in the Dinarides, whence Antić et al. (2014) described three new monospecific genera. However, from the description of these three genera to date, about ten new taxa have been found in caves of the Dinarides, and these monospecific genera will be supplemented in the future (DA pers. obs.). It is worth mentioning that the fauna of the type locality of *B.serbicus* gen. nov. et sp. nov. has been investigated for years, but specimens of this taxon have been found only recently. This leaves no doubt that more trichopolydesmids will be revealed and described from the Balkans in the future.

## Supplementary Material

XML Treatment for Bacillidesmus

XML Treatment for Bacillidesmus filiformis

XML Treatment for Banatodesmus

XML Treatment for Banatodesmus jeanneli

XML Treatment for Napocodesmus

XML Treatment for Napocodesmus florentzae

XML Treatment for Trichopolydesmus

XML Treatment for Trichopolydesmus eremitis

XML Treatment for Balkanodesminus

XML Treatment for Balkanodesminus bulgaricus

XML Treatment for Balkanodesminus dentatus

XML Treatment for Balkanodesminus dentatoides

XML Treatment for Balkanodesminus serbicus

XML Treatment for Rhodopodesmus

XML Treatment for Rhodopodesmus niveus

XML Treatment for Cottodesmus crissolensis

XML Treatment for Haplocookia enghoffi

XML Treatment for Heterocookia tunisiaca
